# Structural basis for mismatch surveillance by CRISPR–Cas9

**DOI:** 10.1038/s41586-022-04470-1

**Published:** 2022-03-02

**Authors:** Jack P. K. Bravo, Mu-Sen Liu, Grace N. Hibshman, Tyler L. Dangerfield, Kyungseok Jung, Ryan S. McCool, Kenneth A. Johnson, David W. Taylor

**Affiliations:** 1 Department of Molecular Biosciences, University of Texas at Austin, Austin, TX USA; 2 Interdisciplinary Life Sciences Graduate Programs, University of Texas at Austin, Austin, TX USA; 3 Center for Systems and Synthetic Biology, University of Texas at Austin, Austin, TX USA; 4 Livestrong Cancer Institutes, Dell Medical School, University of Texas at Austin, Austin, TX USA

**Keywords:** Enzyme mechanisms, Cryoelectron microscopy, DNA metabolism, Genetic engineering

## Abstract

CRISPR–Cas9 as a programmable genome editing tool is hindered by off-target DNA cleavage^1–4^, and the underlying mechanisms by which Cas9 recognizes mismatches are poorly understood^5–7^. Although Cas9 variants with greater discrimination against mismatches have been designed^8–10^, these suffer from substantially reduced rates of on-target DNA cleavage^5,11^. Here we used kinetics-guided cryo-electron microscopy to determine the structure of Cas9 at different stages of mismatch cleavage. We observed a distinct, linear conformation of the guide RNA–DNA duplex formed in the presence of mismatches, which prevents Cas9 activation. Although the canonical kinked guide RNA–DNA duplex conformation facilitates DNA cleavage, we observe that substrates that contain mismatches distal to the protospacer adjacent motif are stabilized by reorganization of a loop in the RuvC domain. Mutagenesis of mismatch-stabilizing residues reduces off-target DNA cleavage but maintains rapid on-target DNA cleavage. By targeting regions that are exclusively involved in mismatch tolerance, we provide a proof of concept for the design of next-generation high-fidelity Cas9 variants.

## Abstract (web summary)

Cryo-electron microscopy structures of Cas9 during mismatch cleavage provide insight into the mechanisms that control off-target effects of Cas9, which will aid in the future design of high-fidelity Cas9 variants with reduced off-target cleavage.

## Main

For therapeutic applications of CRISPR–Cas9, off-target DNA cleavage must be minimized^1–3^. Although a variety of high-fidelity Cas9 variants with improved mismatch discrimination have been developed^7,9^, their enhanced specificity comes at the cost of severely reduced rates of on-target DNA cleavage^5,11^. Mismatches induce alternative Cas9 conformations^12,13^; however, the structures used to guide rational redesign of such variants were bound to on-target DNA and in inactive conformations^14,15^. To understand the molecular mechanisms that govern off-target recognition, here we used kinetic analysis to guide sample preparation for cryo-electron microscopy (cryo-EM) and obtained structural snapshots of Cas9 pre-cleavage activation intermediates in the presence of various guide RNA–DNA target strand (gRNA–TS) mismatches.

## Kinetics of Cas9 on mismatched DNA

**Extended Data Fig. 1:**
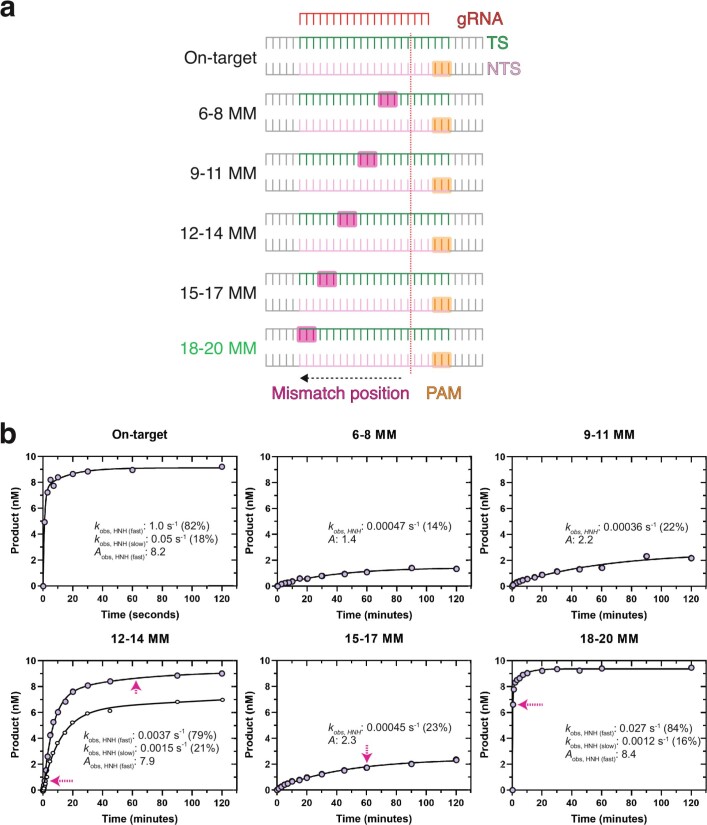
Kinetic basis for mismatch discrimination by Cas9. **a**, Schematic representation of mismatch constructs used for kinetic analysis. **b**, Time course of cleavage of on-target and mismatched DNA (10 nM) by Cas9. Magenta arrows correspond to time-points used to prepare cryo-EM samples. *A*_obs_ corresponds to amplitude of product formed (i.e. total cleavage). For 12–14 MM, target strand cleavage is shown with larger filled circles, while NTS cleavage is given with smaller open circles. For other mismatches we only show target strand cleavage. We previously reported NTS cleavage data for on-target^28^ and 18–20 MM substrates^5^.

We measured the rates of target strand cleavage by Cas9 in the presence of contiguous triple nucleotide mismatches at different positions along the gRNA–TS duplex (Extended Data Fig. 1a, Extended Data Table 1). Compared to rapid on-target cleavage (around 1.0 s^−1^) the well-characterized protospacer adjacent motif (PAM)-distal 18–20 MM^5,9,12,13^ (three mismatches 18–20 bp distal from the PAM) caused a reduction in rate of around 40-fold. Other mismatches (6–8 MM, 9–11 MM and 15–17 MM) resulted in a greater-than-2,000-fold reduction in cleavage rates, with only 20% of the DNA cleaved after 2 h of incubation (Extended Data Fig. 1b).

Notably, the 12–14 MM allowed Cas9 activation but with rates around 10-fold slower than those of the 18–20 MM. Although Cas9 cleavage is markedly slower for both 12–14 MM-and 18–20 MM-containing DNA than for on-target DNA, more than 80% of either substrate was cleaved within an hour of incubation with Cas9. This time frame for off-target cleavage poses problems for genome-editing applications, which typically occur on the time scale of days to weeks^16^.

## Structures of Cas9 with mismatched DNA

**Fig. 1:**
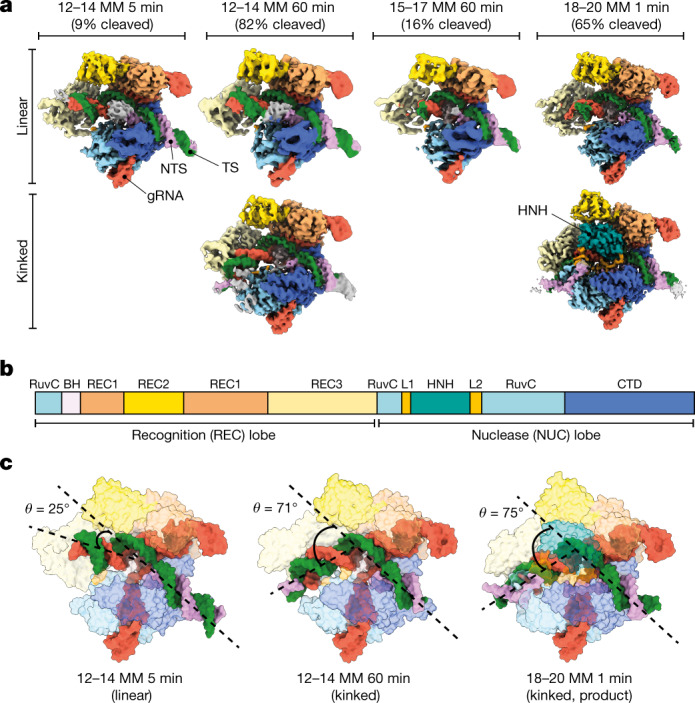
Mismatch-induced Cas9 conformational intermediates. **a**, Cryo-EM reconstructions of Cas9 in complex with various partially mismatched DNA substrates, determined at nominal resolutions ranging from 2.8 to 3.6 Å. Cryo-EM structures are coloured according to the domain map for Cas9. Nucleotides are coloured: target strand (TS), green; NTS, pink; and gRNA, red. The fraction of target strand DNA cleaved by Cas9 containing contiguous triple mismatches at the position and time point used for structural determination is shown above each structure. **b**, Domain organization of SpCas9. CTD, C-terminal domain. **c**, Models of Cas9 in complex with mismatched DNA substrates shown as isosurface representations. The angle between PAM-proximal and PAM-distal duplexes (*θ*) is shown. *θ* is equivalent to around 25º for all linear conformations observed.

**Extended Data Fig. 2:**
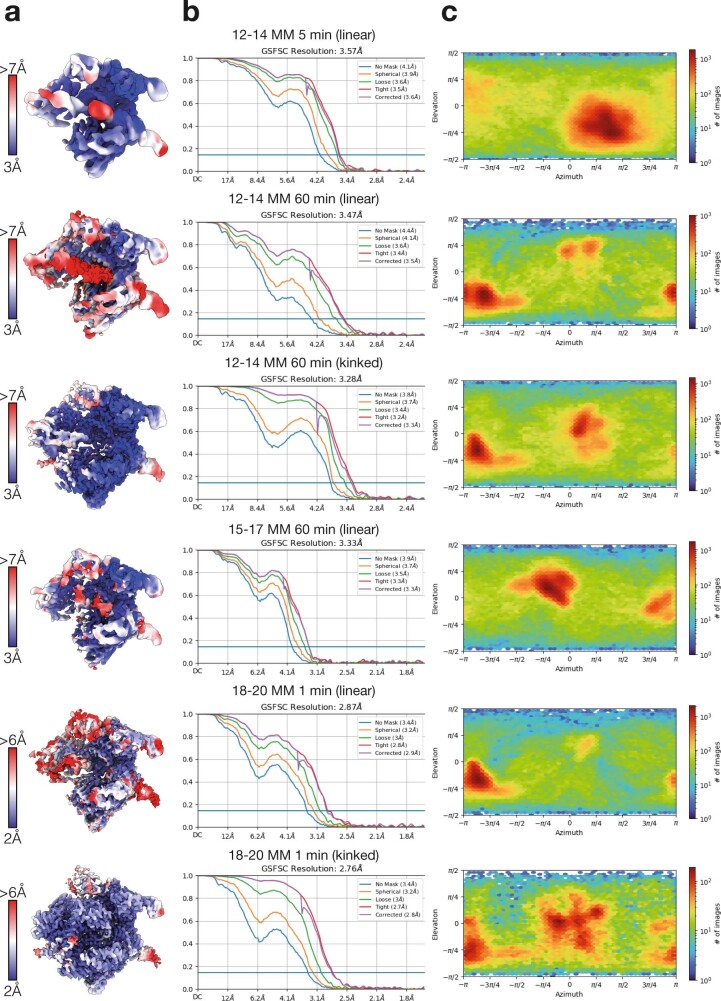
Resolution estimates and orientation distributions of cryo-EM maps. **a**, Unsharpened maps coloured according to local resolution. **b**, Gold-standard FSC curves for cryo-EM reconstructions. Resolutions were estimated at FSC=0.143. **c**, Euler diagrams showing orientation distributions of cryo-EM reconstructions.

To understand the structural basis for Cas9 activation of mismatched DNA, we vitrified Cas9 with 12–14 MM DNA after a 5-min reaction, in which only around 10% of DNA was cleaved (Extended Data Table 2). We determined a cryo-EM structure at a global resolutionof 3.6 Å (Fig. 1a, Extended Data Fig. 2, Extended Data Table 3). The target-strand-cleaving HNH endonuclease domain was not observed, indicating conformational heterogeneity before activation^17,18^. Of note, the distal end of the gRNA–TS duplex was in a linear conformation relative to the PAM-proximal DNA–DNA duplex—a state that differs from previously determined on-target DNA-bound Cas9 structures that depict a kinked duplex (around 70°)^14,18^, although this state is reminiscent of early R-loop formation intermediates^19^.

**Fig. 2:**
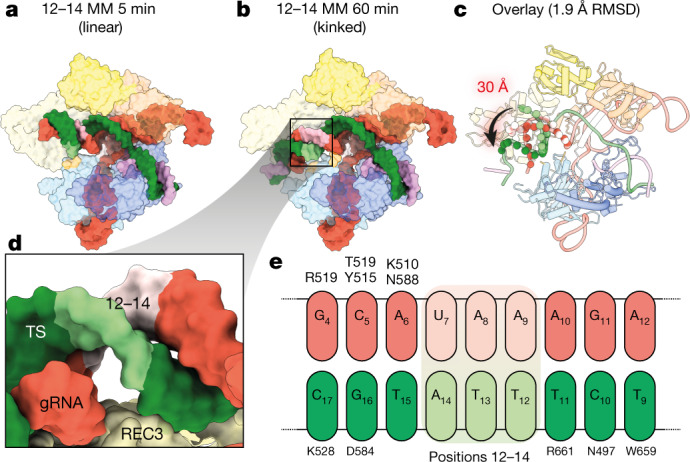
Positions 12–14 of the gRNA–TS duplex occupy a blind spot for REC3 mismatch detection. **a**, **b**, Structures of 12–14 MM at 5 min (**a**) and 1 h (**b**) in linear and kinked conformations, respectively. The position of the 12–14 MM is shown as light green and light pink for the gRNA and the target strand, respectively. Models are shown as isosurface representations. **c**, Conformational change of the PAM-distal gRNA–TS duplex. The Cas9 protein structure is largely unchanged (root-mean-square deviation (RMSD) of less than 2 Å for equivalent C-alpha atoms), but the PAM-distal gRNA–TS duplex end undergoes a 30 Å conformational change, docking with REC3. **d**, Close-up view of positions 12–14, showing that because of the phase of the gRNA–TS duplex, REC3 makes no contacts with these base pairs. **e**, Schematic of interactions between REC3 and positions 9–17 of the gRNA–TS duplex. No interactions occur between Cas9 REC3 and positions 12–14 MM. Position 1 of the duplex is the first base of the target strand that hybridizes with the gRNA spacer.

We then vitrified samples of Cas9 with 12–14 MM DNA after a 1-h incubation in which around 80% of the DNA was cleaved (Fig. 1b). Two distinct conformations were observed: a linear duplex conformation consistent with the 5-min structure of 12–14 MM and the kinked duplex conformation described above (Fig. 1a, c). The Cas9 conformations in the two 12–14 MM structures are identical (Fig. 2), but the PAM-distal gRNA–TS duplex end was shifted by around 30 Å and stably docked with REC3 (Fig. 2c). We propose that the linear duplex conformation corresponds to an early intermediate of Cas9, before HNH rearrangement and docking to cleave the DNA^9,18^. This is supported by recent structural analyses of catalytically dead Cas9 in complex with various R-loop formation intermediates, several of which exhibit linear gRNA–TS duplex conformations that are similar to our linear duplex structures^20^.

Notably, positions 12–14 of the gRNA–TS make no direct contacts with the REC3 domain of Cas9 (Fig. 2). Although positions 9–11 and 15–17 make considerable contacts with REC3, the alignment of the gRNA–TS duplex leaves positions 12–14 without any engagement with this domain (Fig. 2d, e). Because REC3 has a critical role in sensing PAM-distal mismatches^9^, the 12–14 MM is likely to be able to evade mismatch discrimination by REC3 as it is positioned in a blind spot.

We reasoned that mismatches that prevent the PAM-distal gRNA–TS duplex from docking on REC3 would be unable to assume the kinked conformation, leading to considerably reduced DNA cleavage. To test this hypothesis, we determined a structure of Cas9 with 15–17 MM double-stranded DNA (dsDNA) substrate after 1 h of incubation with the enzyme (Fig. 1b). This mismatch inhibits cleavage by Cas9, but still permits DNA binding as measured by high-throughput profiling^21^. We observed only the linear duplex conformation (Fig. 1a, c). These structures support a model in which a linear duplex conformation precedes the canonical kinked duplex conformation that is required for activation, and mismatches that block formation of the kinked conformation escape DNA cleavage by Cas9.

## The 18–20 mismatch supports Cas9 activation

We next sought to understand how certain mismatches can evade Cas9 discrimination to allow more efficient Cas9 activation and DNA cleavage relative to other mismatches. We examined Cas9 after incubation with 18–20 MM DNA at the 1-min time point at which around 65% of the DNA was cleaved (Extended Data Fig. 1b), to determine whether this more tolerated mismatch undergoes the same structural transition as that of 12–14 MM DNA. Consistent with the fraction of product formation, we observed a mixed population of particles including the linear (Fig. 1a, c) and the kinked duplex conformation. In the kinked duplex structure, we observed HNH docked at the target site scissile phosphate, indicating the fully active conformation. This arrangement of HNH is entirely consistent with the previously observed active Cas9 conformation^12,18^. These results suggest that the population of particles showing a linear conformation represents an early intermediate in the pathway, and that the kinking of the gRNA–TS duplex is linked to HNH docking.

**Fig. 3:**
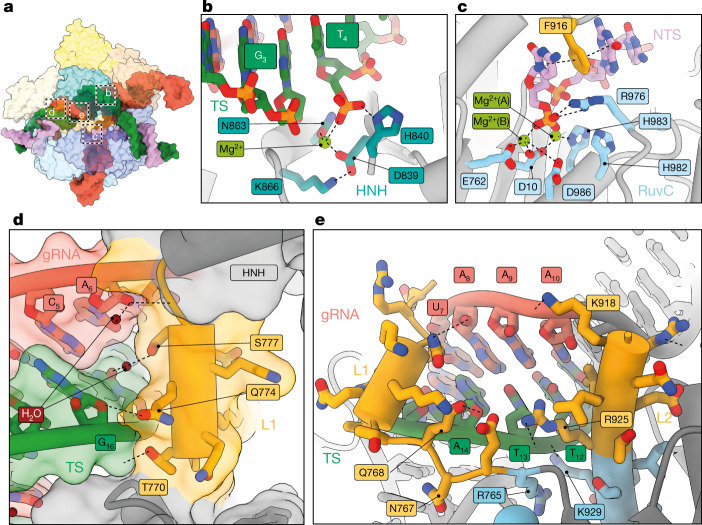
Linkers L1 and L2 mediate the structural transition to the active state. **a**, Overview of the 18–20 MM active conformation. **b**, **c**, Detailed view of HNH (**b**) and RuvC (**c**) active sites. **d**, Docking of the L1 linker helix against the PAM-distal gRNA–TS duplex, shown as an isosurface representation. **e**, Interactions of L1 and L2 regions with the minor groove of the gRNA–TS duplex. HNH extending from L1 and L2 linkers has been removed for clarity and does not interact with this region of the gRNA–TS duplex.

**Extended Data Fig. 3:**
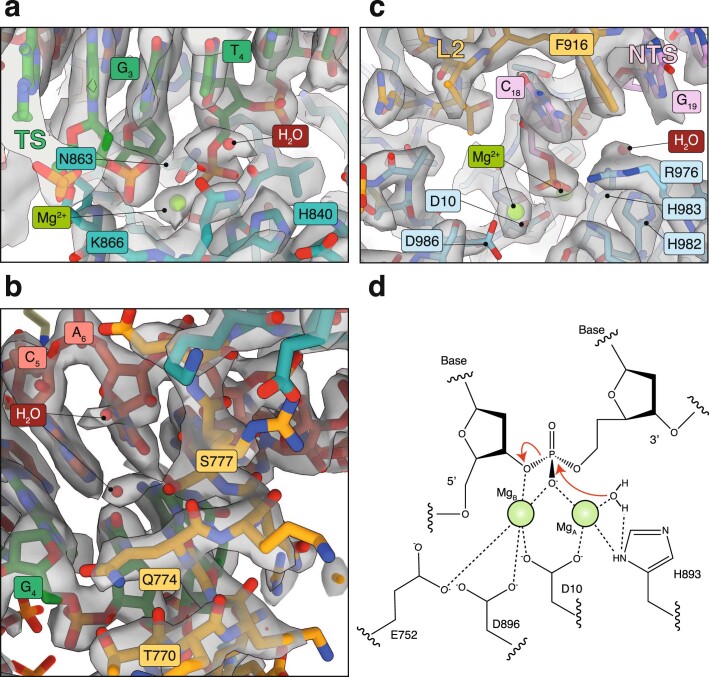
Representative cryo-EM densities for 18–20 MM 1-min kinked (product) structure. **a**, HNH active site, showing cleaved target strand. **b**, L1 linker docked on PAM-distal kinked gRNA–TS duplex. Two water molecules are involved within the network of interactions that stabilize the L1 helix conformation. **c**, RuvC active site, showing cleaved NTS, and positioning of two Mg^2+^ ions. **d**, RuvC DNA cleavage mechanism. This is a typical two-metal-ion mechanism as described in^25^ and agrees with QM/MM simulations for histidine-mediated activation^26^, and the proposed mechanisms of Cas12j and Cas12i^41,42^.

We observed target strand cleavage between nucleotides 3 and 4 (Fig. 3, Extended Data Fig. 3) and non-target strand (NTS) cleavage at the canonical site three bases upstream from the PAM. We report a direct observation of an RuvC active site with the non-target strand bound in the product state (Fig. 3, Extended Data Fig. 3). R986 is in the ‘down’ conformation, stabilizing the two magnesium ions as predicted by molecular dynamics simulations^22^ (Fig. 3), whereas F916 wedges between the −2 and −3 bases through stacking interactions and positions the −3 position within the RuvC active site. These observations are in agreement with previous structural and mutagenesis studies^23,24^. Our structure suggests a histidine-mediated catalytic mechanism, consistent with two-metal-ion-dependent catalysis^25^ and supported by quantum-classical simulations^26^. Furthermore, our product state reveals that the two Mg^2+^ ions are around 4.2 Å from each other, in agreement with the product state of the histidine-mediated mechanism (Extended Data Fig. 3).

**Extended Data Fig. 4:**
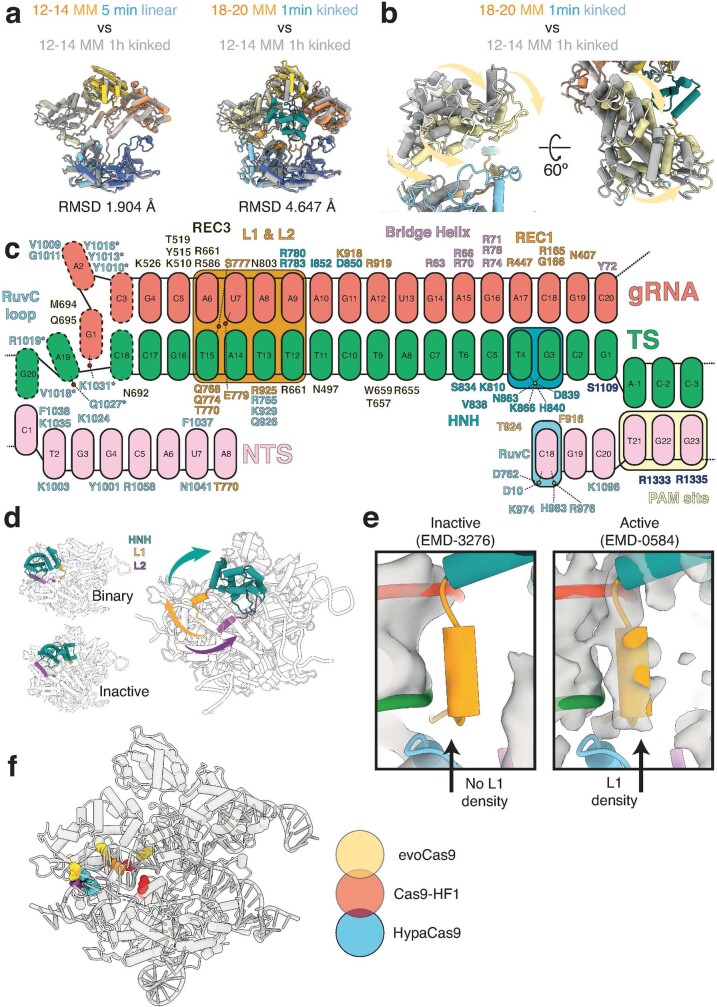
Structural analysis of Cas9. **a**, Left, comparison of Cas9 protein only between 12–14 MM 60 min linear (colour) and 12–14 MM 1-h kinked (grey) models. Right, comparison of Cas9 protein only active conformation (18–20 MM 1 min linear, colour) and kinked pre-active (12–14 MM 60 min kinked, grey) models. While there is no significant conformational change associated between transition from linear to kinked pre-active (root-mean standard deviation (RMSD) between equivalent Cα atoms of 1.904 Å), the change from kinked pre-active to active conformations is associated with a larger conformational change (4.647 Å, most of which occurs within the REC3 domain). **b**, Close-up view of REC3 conformational changes that occur upon activation, as viewed from one angle. REC3 moves forwards towards the kinked duplex by ~15 Å upon activation and HNH repositioning. **c**, Schematic representation of Cas9–nucleic acid contacts in the context of 18–20 MM. Residues mutated in SuperFi-Cas9 are denoted by an asterisk. **d**, Conformations of HNH domain (green) and L1 (gold) and L2 (purple) linkers in the context of Cas9 binary complex (i.e. with gRNA, PDB 4ZT0), Cas9–gRNA complex bound to dsDNA in an inactive conformation (PDB 5F9R), and in the active Cas9 18–20 MM structure presented in this work. Upon activation, HNH is repositioned at the target strand cleavage site, driven by large conformational changes in the L1 and L2 linkers. **e**, Comparison with the active Cas9 18–20 MM structure presented in this work and previously determined cryo-EM maps (transparent grey) of inactive (left, EMD-3276^14^) and active (right, EMD-0584^18^) Cas9 bound to on-target dsDNA. The inactive Cas9 has no density for L1 helix at the kinked distal-docked gRNA–TS site, whereas there is clear density for L1 at this site in the active Cas9 cryo-EM map. **f**, Mapping of residues mutated to alanine in selected high-fidelity Cas9 variants. EvoCas9 (yellow) – M495, Y515, R661, K526. Cas9-HF1 (red) – N497, R661, Q695, Q926. HypaCas9 (blue) – N692, M694, Q695, H698. Residues shared between Cas9-HF-1 and either EvoCas9 or HypaCas9 are shown as orange and purple, respectively.

The fully active configuration requires marked conformational rearrangements, including an approximately 140° rotation of the HNH domain from the inactive state. Furthermore, our structures reveal the molecular mechanisms that underlie this rearrangement. The L1 and L2 linker domains tether HNH to the rest of Cas9 and are often missing from crystal structures, presumably owing to their intrinsic flexibility. However, in our active structure, we observe high-quality density for both L1 and L2. Notably, the L1 helix docks against the minor groove of the PAM-distal gRNA–TS duplex and forms an extended network of interactions, including multiple water-mediated hydrogen bonds with both strands (Fig. 3). As L1 docks on the minor groove, these interactions are gRNA–TS structure-specific rather than sequence-specific and can only occur when the PAM-distal duplex end is in the kinked conformation. This provides a structural basis for our observation that the kinked duplex conformation is an intermediate that precedes Cas9 activation and DNA cleavage. Comparisons of our model with Cas9 structures in inactive (Electron Microscopy Data Bank (EMDB) code EMD-3276) and active (EMD-0584) conformations confirmed that L1 docking against the gRNA–TS duplex is correlated with HNH rearrangement and Cas9 activation (Extended Data Fig. 4). Furthermore, our observation of L1 and L2 ‘locking’ HNH in an active conformation is supported by the slow rate of dissociation of Cas9 from target DNA after cleavage^27^.

Residue F916 stabilizes the NTS and is within the L2 linker domain; however, within the inactive Cas9 conformation, L2 is positioned more than 20 Å away from the RuvC active site. L1-facilitated positioning of HNH on the target strand enables relocation of L2, which in turn enables positioning of the NTS within the RuvC active site (Extended Data Fig. 4). This mechanism provides a structural explanation for the observed coupling of target strand and NTS cleavage, in which HNH docking precedes alignment of the NTS at the RuvC site for cleavage^5,28^. The HNH and RuvC cleavage reactions appear to occur simultaneously because the alignment is rate-limiting.

Although previous studies have noted the importance of L1 docking onto the gRNA–TS duplex for HNH repositioning^23,29^, our observation that a linear gRNA–TS duplex conformation induced by PAM-distal mismatches precludes L1 docking provides a structural explanation for why certain PAM-distal mismatched substrates are able to bind Cas9, while not triggering rapid DNA cleavage^21^.

## The 18–20 mismatch reorders an RuvC loop

**Fig. 4:**
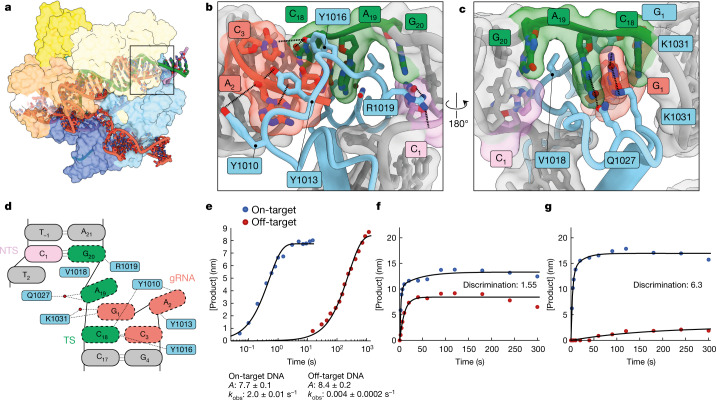
Stabilization of distorted 18–20 MM by the RuvC domain and improved fidelity of SuperFi-Cas9. **a**, Overall structure of the 18–20 MM active conformation viewed from the back. **b**, **c**, Magnified views of Cas9 interacting with the distal end of the duplex. Flipped gRNA base position 2 is accommodated by stacking interactions and hydrogen bonding with RuvC tyrosine side-chains, whereas a network of interactions (including a water-mediated hydrogen bond) stabilizes the stretched target strand configuration, which allows TS(20) to resume base-pairing with the NTS. **d**, Schematic of distorted PAM-distal gRNA–TS duplex. Red circles correspond to water molecules. **e**, Kinetics of on-target and off-target (18–20 MM) Mg^2+^-initiated cleavage by the 7-D Cas9 mutant (SuperFi-Cas9). **f**, **g**, Cleavage competition assay for wild-type Cas9 (**f**) and SuperFi-Cas9 (**g**). 25 nM of either Cas9 variant was mixed with 50 nM of each substrate and the cleaved DNA product was monitored. Discrimination in favour of the on-target DNA is defined by the ratio of amplitudes for on-target and off-target product formed.

**Extended Data Fig. 5:**
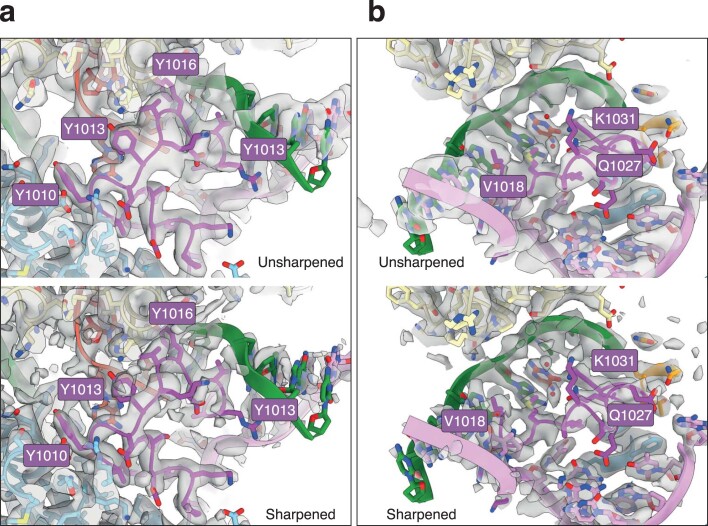
Representative cryo-EM density for the RuvC loop. Two different views are shown (**a**, **b**). Unsharpened and B-factor sharpened maps are shown for each view with the RuvC loop shown as dark magenta. Key residues involved in stabilizing this distorted conformation are labelled.

The 18–20 MM contains an unusual duplex conformation at the site of the mismatch. The C:C mismatch at position 18 on the target strand, TS(18), is stabilized by stacking interactions with adjacent Watson–Crick base pairs. However, the gRNA is otherwise distorted with gRNA position 2 (gRNA(2)) flipped out by around 180º so that gRNA(1) then intercalates between TS(19) and TS(20). TS(19) participates in water-mediated hydrogen bonds to Q1027, and TS(20) resumes base-pairing with NTS (Fig. 4, Extended Data Fig. 5).

**Extended Data Fig. 6:**
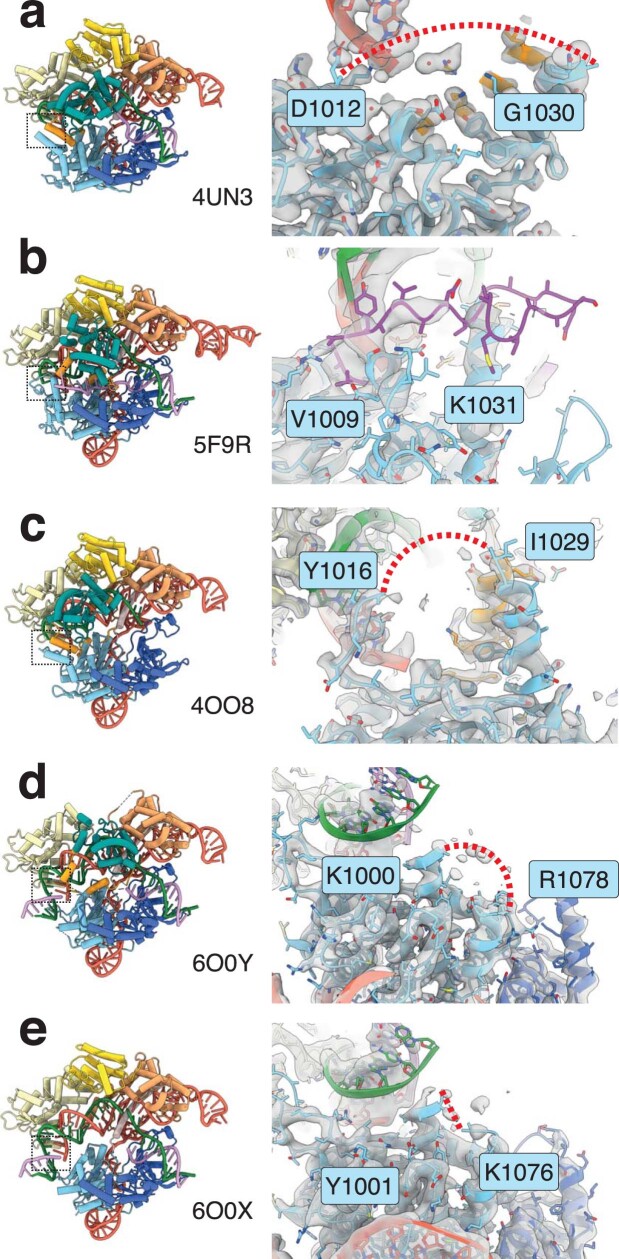
RuvC loop in on-target SpCas9 structures. **a**, On-target inactive Cas9 bound to dsDNA (PDB 4UN3)^15^. RuvC loop is missing between 1013–1029. **b**, On-target inactive (primed – HNH rearranged and adjacent to target strand scissile phosphate) Cas9 bound to dsDNA (PDB 5F9R)^14^. RuvC loop has been built primarily as alanine ‘stub’ residues, but electron density is very poor and diffuse for this region. **c**, On-target inactive Cas9 bound to dsDNA (PDB 4OO8)^43^. RuvC loop is missing between 1017–1028. **d**, On-target active Cas9 bound to dsDNA in postcatalysis state^18^. RuvC loop is missing between 1001–1077. **e**, On-target active Cas9 bound to dsDNA in product state^18^. RuvC loop is missing between 1000–1075. In **a**–**c**, electron density is displayed as a grey surface, and in **d**, **e** cryo-EM density is shown as a grey surface. In all structures, missing residues are depicted as a red dashed line with the RuvC loop in **b** shown as magenta. Position of RuvC loop is denoted by a black dashed box in the left panel for each model.

**Extended Data Fig. 7:**
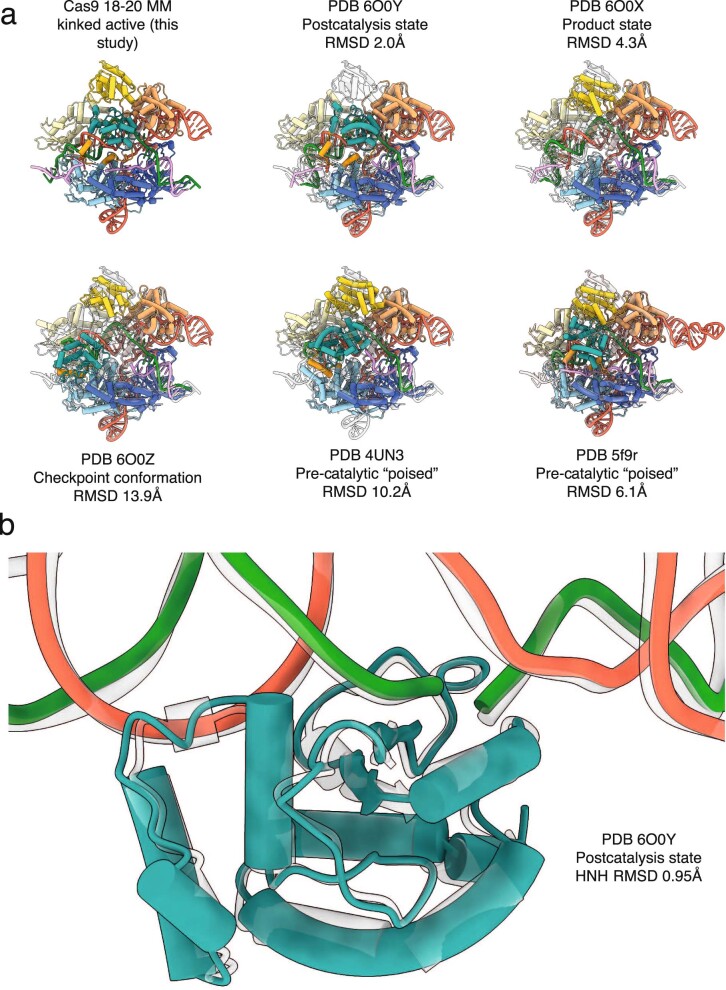
Comparison of Cas9 with previous structures. **a**, Comparison of 18–20 MM kinked product state Cas9 with a selection of previously determined structures. RMSD between equivalent C-alpha atoms is shown. **b**, Alignment of HNH from the 18–20 MM kinked product state presented here (transparent grey) and the previously determined ‘post-catalysis’ state (PDB 6O0Y). The catalytically competent HNH conformation between these two structures is highly similar.

This unusual nucleic acid conformation is stabilized by RuvC and appears to facilitate the binding of this mismatch. The residues within RuvC that contact and stabilize this distorted configuration are absent in previous on-target structures^14,15,18,30^ (Extended Data Fig. 6), despite the overall similarity between our model and a previously determined active on-target Cas9 (Extended Data Fig. 7). This indicates that these resolved RuvC residues are involved only in mismatch binding and not in on-target activation (Fig. 4). Although this mechanism to accommodate certain mismatches may provide an essential mechanism for bacteria to restrict phage variants, it is counterproductive for the use of Cas9 in gene editing.

Previous rationally engineered variants ‘hyper-accurate Cas9’ (HypaCas9; N692A, M694A, Q695A and H698A mutations) and ‘high-fidelity Cas9’ (Cas9-HF1; N467A, R661A, Q695A and Q926A mutations) achieve somewhat higher fidelity at the expense of up to 100-fold reduced efficiency of on-target DNA cleavage^5,8,9^. The mutated residues are mainly located within the REC3 domain and make numerous interactions only with the kinked duplex end. Therefore, by abolishing interactions between REC3 and the PAM-distal duplex, these high-fidelity variants reduce the capacity of Cas9 to stabilize the kinked duplex configuration that is required for the docking of L1, and thereby reduce HNH repositioning and cleavage activity. Our data provide a structural explanation for why these high-fidelity Cas9 variants reduce the activation of Cas9^9^ by off-target substrates, but also reduce on-target Cas9 activity.

To test the role of this loop for mismatch stabilization, we designed a 7-D mutant (in which all seven of the stabilizing residues in Fig. 4b are mutated to aspartic acid) and tested the effects of this mutant on DNA cleavage. Although this 7-D mutant cleaved on-target DNA at a similar rate to wild-type *Streptococcus pyogenes* Cas9 (SpCas9) (2 s^−1^), we observed that cleavage of 18–20 MM DNA was 500-fold slower (0.004 s^−1^) (Fig. 4e). This indicates that this loop is critical for stabilizing the distorted mismatch-induced PAM-distal duplex conformation, thereby allowing the duplex to adopt the kinked conformation that is prerequisite for Cas9 activation. We refer to our designed high-fidelity variant that retains wild-type on-target cleavage rates as ‘SuperFi-Cas9’.

Because enzyme specificity is a kinetic phenomenon that is not determined solely by the rates of the chemical reaction, we performed a direct competition assay, in which on-target and off-target (18–20 MM) dsDNA substrates were mixed simultaneously with enzyme and cleavage was monitored over time. Although wild-type Cas9 showed some preference for on-target substrates (a 1.55-fold specificity ratio favouring the on-target over 18–20 MM off-target DNA), SuperFi-Cas9 showed rapid cleavage of on-target DNA and minimal cleavage of 18–20 MM DNA (6.3-fold preference for on-target DNA) (Fig. 4f, g). The ability to discriminate between on- and off-target DNA substrates without compromising DNA cleavage efficiency appears to be unique to SuperFi-Cas9^11^. Although further studies are needed to fully define the kinetic basis for the change in discrimination, our current data constitute a proof of concept and provide a rationale for engineering improved variants of Cas9 using our structure.

## Discussion

**Fig. 5:**
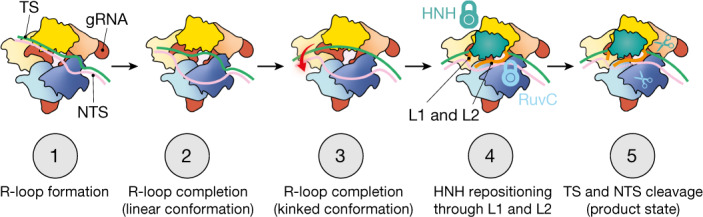
Model for Cas9 activation. During R-loop propagation (step 1), the gRNA–TS duplex adopts a linear conformation. After R-loop completion, the PAM-distal end of the linear duplex is captured by REC3 (steps 2 and 3). Mismatches in the PAM-distal region appear to prevent REC3 docking and thereby block subsequent steps of Cas9 activation. Once the kinked R-loop conformation has been formed, L1 and L2 linkers use the gRNA–TS duplex as a scaffold to position the HNH domain at the scissile phosphate of the target strand and to position the NTS in the RuvC site (step 4), which enables Cas9 to make a double-strand break (step 5). According to this model, mutations in the RuvC loop (corresponding to SuperFi-Cas9) inhibit formation of the kinked conformation and subsequent cleavage of the gRNA–TS duplex with mismatches at the PAM-distal end.

Through kinetics-guided structural determination, we have described a gRNA–TS duplex conformational intermediate that precedes Cas9 activation (Fig. 5). Notably, we observe that the well-characterized and widespread off-target cleavage of DNA containing mismatches at the extreme PAM-distal end (positions 18–20 (refs.^5,9,12,31,32^)) is attributed to a unique mechanism that stabilizes a highly distorted duplex conformation, involving a domain loop in RuvC that penetrates the duplex. This region is missing in previously determined structures of Cas9, which suggests that it has a role solely in mismatch tolerance at these positions. Our results provide molecular insights into the underlying structural mechanisms that govern off-target effects of Cas9, and provide a molecular blueprint for the design of next-generation high-fidelity Cas9 variants that reduce off-target DNA cleavage while retaining efficient cleavage of on-target DNA.

## Methods

### Protein expression and purification

SpCas9 was expressed and purified as described previously^5^.

### Nucleic acid preparation

DNA duplexes (55 nt) were prepared from PAGE-purified oligonucleotides synthesized by Integrated DNA Technologies. DNA duplexes used in cleavage assays were prepared by mixing 6-FAM- or Cy3-labelled target strands with unlabelled non-target strands at a 1:1.15 molar ratio in annealing buffer (10 mM Tris-HCl pH 8, 50 mM NaCl and 1 mM EDTA), heating to 95 °C for 5 min, then cooling to room temperature over the course of 1 h. The sgRNA was purchased from Synthego and annealed in annealing buffer using the same protocol as for the duplex DNA substrates. The sequences of the synthesized oligonucleotides, including the positions of mismatches, are listed in Extended Data Table 1.

### Kinetics

#### Buffer composition for kinetic reactions

Cleavage reactions were performed in 1× cleavage buffer (20 mM Tris-Cl, pH 7.5, 100 mM KCl, 5% glycerol and 1 mM DTT) at 37 °C.

#### DNA cleavage kinetics

The reaction of Cas9 with on- and off-target DNA was performed by preincubating Cas9.gRNA (28 nM active-site concentration of Cas9, 100 nM gRNA) with 10 nM DNA with a 6-FAM label on the target strand in the absence of Mg^2+^. The reaction was initiated by adding Mg^2+^ to 10 mM, then stopped at various times by mixing with 0.3 M EDTA (Extended Data Fig. 1). Products of the reaction were resolved and quantified using an Applied Biosystems DNA sequencer (ABI 3130xl)^33^. Data were fit using either a single or a double-exponential equation, as shown below.

Single exponential equation:

1 $$Y={A}_{1}{{\rm{e}}}^{-\lambda 1t}+C$$

in which *Y* represents the concentration of the cleavage product, *A*_1_ represents the amplitude and *λ*_1_ represents the observed decay rate (eigenvalue). The half-life was calculated as *t*_1/2_ = ln(2)/*λ*_1_.

Double exponential equation:

2 $$Y={A}_{1}{{\rm{e}}}^{-\lambda 1t}\,{A}_{2}{{\rm{e}}}^{-\lambda 2t}+C$$

in which *Y* represents the concentration of the cleavage product, *A*_1_ represents the amplitude and *λ*_1_ represents the observed rate for the first phase. *A*_2_ represents the amplitude and *λ*_2_ represents the observed rate for the second phase.

### Kinetic competition assay

Enzyme specificity is a kinetic phenomenon that is a function of all steps leading up to and including the first largely irreversible step in the pathway and it is common for mutants to introduce a change in specificity determining steps^34^. Therefore, we designed an assay to monitor relative rates of cleavage for on- and off-target DNA when the enzyme was presented with both substrates simultaneously. The competition assay was performed by mixing a solution of 25 nM (active site concentration) Cas9 and 100 nM sgRNA, in the presence of 10 mM Mg^2+^, with 50 nM on-target DNA and 50 nM off-target DNA, in which the DNA contained a 5′-6-FAM label or a 5′-Cy3 label on the target or off-target DNA, respectively. Time points were collected by mixing with 0.3 M EDTA and reaction products were resolved and quantified by capillary electrophoresis, as described above. On-target cleavage data were fit to a single exponential function and off-target cleavage data were fit to a double exponential function. Discrimination was calculated as the ratio of the total amplitude of on-target cleavage divided by the amplitude for off-target cleavage to derive the relative specificity constants for the on-target DNA compared to the off-target DNA.

### Cryo-EM sample preparation, data collection and processing

Cas9 in complex with various mismatched DNA substrates was frozen at different time points, on the basis of kinetic analysis (Extended Data Fig. 1). A non-productive mismatch complex (15–17 MM, 1 h); a slow productive mismatch (12–14) at early (5 min) and late (1 h) time points; and a fast productive mismatch (18–20, 1 min) were chosen. MDCC-Cas9 was used for structure determination to couple structural analysis with ongoing kinetic studies monitoring changes in fluorescence. It has previously been shown that the kinetics of MDCC-Cas9 were indistinguishable from those of wild-type enzyme^5^. The cleavage reaction was triggered by mixing 10 µM DNA duplex preincubated with 10 mM MgCl_2_ and 8 µM MDCC-labelled Cas9: 8 µM gRNA was preincubated with 10 mM MgCl_2_, in reaction buffer (19 mM Tris-Cl, pH 7.5, 95 mM KCl, 4.75% glycerol and 5 mM DTT) at a 1:1 ratio. Four microlitres of sample was applied to glow-discharged holey carbon grids (C-flat 2/2, Protochips), blotted for 1 s with a blot force of 4 and rapidly plunged into liquid nitrogen-cooled ethane using an FEI Vitrobot MarkIV. Reactions were quenched through vitrification.

Data were collected on an FEI Titan Krios cryo-electron microscope equipped with a K3 Summit direct electron detector (Gatan). Images were recorded with SerialEM^35^ with a pixel size of 1.1 Å for 12–14 MM datasets, and 0.81 Å for 18–20 MM and 15–17 MM datasets, over a defocus range of −1.5 to −2.5 µm. During collection of the 12–14 MM 5-min time-point dataset, a preferred orientation was observed. To ameliorate this, a second dataset was collected at 30° tilt. Movies were recorded at 13.3 electrons per pixel per s for 6 s (80 frames) to give a total dose of 80 electrons per pixel. CTF correction, motion correction and particle picking were performed in real-time using cryoSPARC Live. Further data processing was performed with cryoSPARC v.3.2^36^.

Multiple rounds of 3D classification within cryoSPARC yielded reconstructions of six distinct Cas9 complexes at resolutions ranging from 2.7 to 3.6 Å (Extended Data Table 3). To aid the separation of multiple Cas9 conformational states from within the same dataset, 3D variability analysis was performed within CryoSPARC. First and last frames from suitable eigenvector trajectory were then used as references for heterogeneous refinement (that is, reference-based 3D classification), and particles from resulting classes were refined using non-uniform refinement and used for final reconstructions^37^. Active Cas9 (Protein Data Bank (PDB) code: 6O0X) was rigid-body fitted into each map using ChimeraX^38^. Regions of the model not present in a given map were truncated, and flexible fitting was performed using Namdinator^39^. Further modelling was performed using Isolde^40^, and the models were ultimately subjected to real-space refinement as implemented in PHENIX.

### Reporting summary

Further information on research design is available in the Nature Research Reporting Summary linked to this paper.

## Online content

Any methods, additional references, Nature Research reporting summaries, source data, extended data, supplementary information, acknowledgements, peer review information; details of author contributions and competing interests; and statements of data and code availability are available at 10.1038/s41586-022-04470-1.

### Supplementary information

Reporting Summary

Peer Review File

## Figures and Tables

**Extended Data Table 1:**
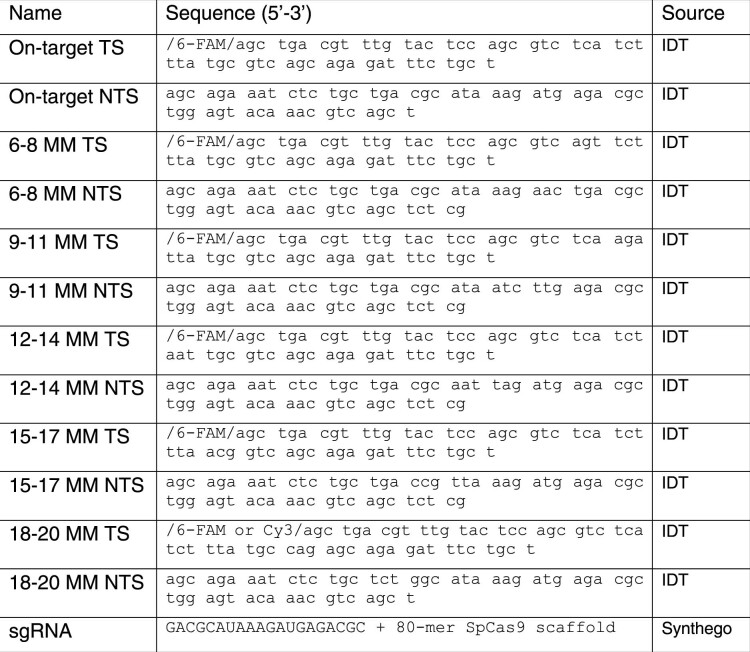
List of nucleotide sequences used in the study

**Extended Data Table 2:**
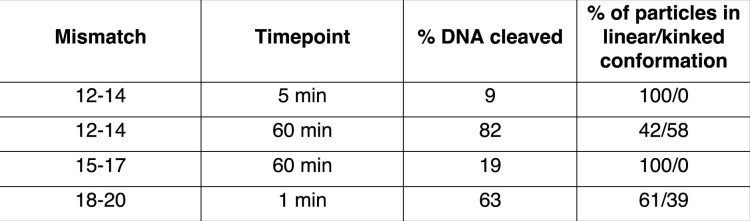
Correlation between fraction of DNA cleaved and fraction of cryo-EM particles in linear or kinked duplex conformations

**Extended Data Table 3:**
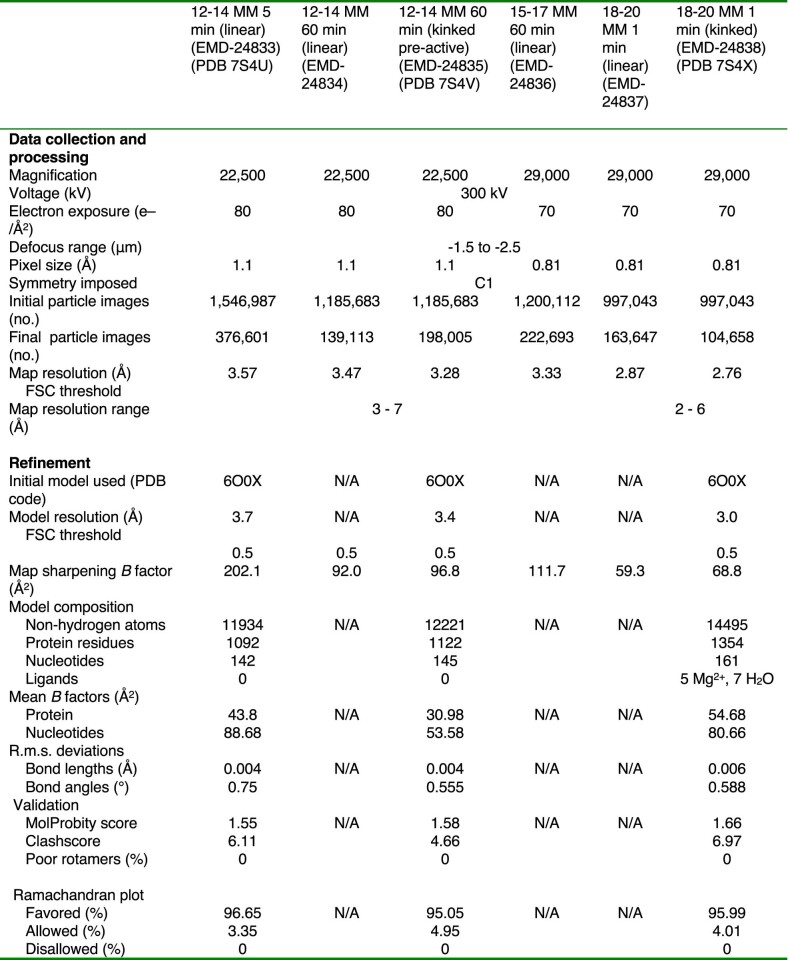
Cryo-EM data collection, refinement and validation statistics

## Data Availability

The structures of 12–14 MM 5 min, 12–14 MM 60-min linear and 18–20 MM 1-min kinked active, and their associated atomic coordinates, have been deposited into the EMDB and the PDB with EMDB accession codes EMD-24833, EMD-24835 and EMD-24838 and PDB accession codes7S4U, 7S4V and 7S4X, respectively. Maps of 12–14 MM 60-min linear, 15–17 MM 60-min linear and 18–20 1-min linear have been deposited into the EMDB with accession codes EMD-23834, EMD-24836 and EMD-24837, respectively.
