# Publisher Correction: Structural basis for mismatch surveillance by CRISPR–Cas9

**DOI:** 10.1038/s41586-022-04655-8

**Published:** 2022-03-22

**Authors:** Jack P. K. Bravo, Mu-Sen Liu, Grace N. Hibshman, Tyler L. Dangerfield, Kyungseok Jung, Ryan S. McCool, Kenneth A. Johnson, David W. Taylor

**Affiliations:** 1 Department of Molecular Biosciences, University of Texas at Austin, Austin, TX USA; 2 Interdisciplinary Life Sciences Graduate Programs, University of Texas at Austin, Austin, TX USA; 3 Center for Systems and Synthetic Biology, University of Texas at Austin, Austin, TX USA; 4 Livestrong Cancer Institutes, Dell Medical School, University of Texas at Austin, Austin, TX USA

**Keywords:** Enzyme mechanisms, Cryoelectron microscopy, DNA metabolism, Genetic engineering

Correction to: *Nature* 10.1038/s41586-022-04470-1 Published online 2 March 2022

In the version of this article initially published, Extended Data Fig. 4 was an inadvertent duplicate of Extended Data Fig. 2. The image has been replaced in the HTML and PDF versions of the article

