# Supplementary material for: Structural basis for mismatch surveillance by CRISPR–Cas9
Source: Nature. 2022 Mar 2;603(7900):343–7. doi: 10.1038/s41586-022-04470-1 (PMC8907077; doi:10.1038/s41586-022-04470-1)
Supplement: Supplementary file 1 — Reporting Summary [file 41586_2022_4470_MOESM1_ESM.pdf]

nature portfolio

Corresponding author(s): Kenneth A. Johnson and David W. Taylor

Last updated by author(s): Jan 10, 2022

# Reporting Summary

Nature Portfolio wishes to improve the reproducibility of the work that we publish. This form provides structure for consistency and transparency in reporting. For further information on Nature Portfolio policies, see our Editorial Policies and the Editorial Policy Checklist.

## Statistics

For all statistical analyses, confirm that the following items are present in the figure legend, table legend, main text, or Methods section.

| n/a | Confirmed | |
|---|---|---|
| ☐ | ☒ | The exact sample size (*n*) for each experimental group/condition, given as a discrete number and unit of measurement |
| ☐ | ☒ | A statement on whether measurements were taken from distinct samples or whether the same sample was measured repeatedly |
| ☒ | ☐ | The statistical test(s) used AND whether they are one- or two-sided<br>*Only common tests should be described solely by name; describe more complex techniques in the Methods section.* |
| ☒ | ☐ | A description of all covariates tested |
| ☒ | ☐ | A description of any assumptions or corrections, such as tests of normality and adjustment for multiple comparisons |
| ☒ | ☐ | A full description of the statistical parameters including central tendency (e.g. means) or other basic estimates (e.g. regression coefficient) AND variation (e.g. standard deviation) or associated estimates of uncertainty (e.g. confidence intervals) |
| ☒ | ☐ | For null hypothesis testing, the test statistic (e.g. *F*, *t*, *r*) with confidence intervals, effect sizes, degrees of freedom and *P* value noted<br>*Give P values as exact values whenever suitable.* |
| ☒ | ☐ | For Bayesian analysis, information on the choice of priors and Markov chain Monte Carlo settings |
| ☒ | ☐ | For hierarchical and complex designs, identification of the appropriate level for tests and full reporting of outcomes |
| ☒ | ☐ | Estimates of effect sizes (e.g. Cohen's *d*, Pearson's *r*), indicating how they were calculated |

*Our web collection on statistics for biologists contains articles on many of the points above.*

## Software and code

Policy information about availability of computer code

| | |
|---|---|
| Data collection | Data was collected on a Titan Krios equipped with a K3 direct electron detector. SerialEM 3.8 (ref 38) was used for automated cryo-EM data collection. |
| Data analysis | Cryo-EM data acquisition was monitored by on-the-fly pre-processing in cryoSPARC Live. Data were further processed using cryoSPARC v3.2 (ref 39). Model building and refinement of the structures were performed using Map to Model in PHENIX v1.18rc5 as well as Namdinator (ref 42). Models were also manually built in Isolde (ref 43). Model validation was performed using MolProbity as implemented within PHENIX. Visualization was performed using ChimeraX v1.0 (ref 41). |

For manuscripts utilizing custom algorithms or software that are central to the research but not yet described in published literature, software must be made available to editors and reviewers. We strongly encourage code deposition in a community repository (e.g. GitHub). See the Nature Portfolio guidelines for submitting code & software for further information.

## Data

Policy information about availability of data

All manuscripts must include a data availability statement. This statement should provide the following information, where applicable:

- Accession codes, unique identifiers, or web links for publicly available datasets
- A description of any restrictions on data availability
- For clinical datasets or third party data, please ensure that the statement adheres to our policy

The structures of 12-14MM 5 min, 12-14MM 60 min linear and 18-20MM 1 min kinked active have been and their associated atomic coordinates have been deposited into the Electron Microscopy Data Bank (EMDB) and Protein Data Bank (PDB) with accession codes EMD-24833, EMD-24835, EMD-24838 and PDB codes 7S4U, 7S4V and 7S4X, respectively. Maps of 12-14MM 60 min linear, 15-17MM 60 min linear and 18-20 1 min linear have been deposited into the Electron

Microscopy Data Bank (EMDB) with accession codes EMD-23834, EMD-24836 and EMD-24837, respectively. All materials are available upon request from Kenneth A. Johnson and David W. Taylor.

# Field-specific reporting

Please select the one below that is the best fit for your research. If you are not sure, read the appropriate sections before making your selection.

☒ Life sciences ☐ Behavioural & social sciences ☐ Ecological, evolutionary & environmental sciences

For a reference copy of the document with all sections, see nature.com/documents/nr-reporting-summary-flat.pdf

# Life sciences study design

All studies must disclose on these points even when the disclosure is negative.

| | |
|---|---|
| Sample size | A total of between 1,848 - 2,172 micrographs were collected for each dataset. Each dataset contained at least 997,043 particles, and at least 104,658 were used for the final reconstruction. These are typical image numbers for cryo-EM datasets to obtain high resolution reconstructions. |
| Data exclusions | 2D and 3D classification procedures were used to exclude damaged and 'bad' particles. This is standard practice in cryo-EM and is necessary in order to obtain homogeneous high resolution cryoEM structures. |
| Replication | Cryo-EM datasets were collected with multiple samples in separate imaging sessions. Linear gRNA:TS conformations present within all 4 datasets were nearly identical. |
| Randomization | No randomization was performed. Randomization is not relevant to this study because the work did not involve human subjects or live animals. |
| Blinding | No blinding was performed. Blinding is not relevant to this study because the work did not involve human subjects or live animals. |

# Reporting for specific materials, systems and methods

We require information from authors about some types of materials, experimental systems and methods used in many studies. Here, indicate whether each material, system or method listed is relevant to your study. If you are not sure if a list item applies to your research, read the appropriate section before selecting a response.

## Materials & experimental systems

| n/a | Involved in the study | |
|---|---|---|
| ☒ | ☐ | Antibodies |
| ☒ | ☐ | Eukaryotic cell lines |
| ☒ | ☐ | Palaeontology and archaeology |
| ☒ | ☐ | Animals and other organisms |
| ☒ | ☐ | Human research participants |
| ☒ | ☐ | Clinical data |
| ☒ | ☐ | Dual use research of concern |

## Methods

| n/a | Involved in the study | |
|---|---|---|
| ☒ | ☐ | ChIP-seq |
| ☒ | ☐ | Flow cytometry |
| ☒ | ☐ | MRI-based neuroimaging |
