# Supplementary material for: Structural basis for mismatch surveillance by CRISPR–Cas9
Source: Nature. 2022 Mar 2;603(7900):343–7. doi: 10.1038/s41586-022-04470-1 (PMC8907077; doi:10.1038/s41586-022-04470-1)
Supplement: Supplementary file 2 — Peer Review File [file 41586_2022_4470_MOESM2_ESM.docx]

**Peer Review File**

**Manuscript Title: Structural basis for mismatch surveillance by CRISPR-Cas9**

**Reviewer Comments & Author Rebuttals**

**Reviewer Reports on the Initial Version:**

Referees' comments:

Referee #1:

CRISPR/Cas9 as a programmable genome editing tool has been hindered by off-target DNA cleavage. Bravo et al. performed enzyme kinetics to investigate the impact of mismatches within different regions on SpCas9 domain rearrangement during R-loop extension. The authors determined a series of structures with mismatches at different positions, including 12-14 mm, 15-17 mm and 18-20 mm. These structures provide snapshots of the conformation of SpCas9 bound to the mismatched target DNA. The authors also captured the RuvC catalytically-competent conformation with the non-target strand bound in the active site. Together, this structural study provides new conformational change of Cas9 for the activation, and may provide hints for the rational design of Super Cas9 with higher fidelity, i.e., disrupting conformations that bind to mismatched targets while avoiding disrupting interactions to stabilize intermediate states that bind to the correct target DNA.

Major:

1. In the 18-20 mm structure, the loop of residues 1009-1016 in the RuvC domain interacts with the mismatch residues to stabilize the unusual nucleic acid conformation. The authors found that these interactions were only present in the 18-20 mm complex and not in the target complex. How do mutations in these residues that interact with mismatched nucleotides affect the cleavage of mismatched targets and target DNA by Cas9, respectively? Do these amino acid mutations increase the accuracy of Cas9?

2. It is easy to understand that the mismatches at the PAM distal region have less effect than at other positions along the sgRNA-TS duplex. Why does 15-17 mm have lower activity than 12-14 mm? What is the detailed conformation of the mismatched 12-14 and 15-17 base pairs?

3. The Cas9 in the complex of Cas9-12-14 mm DNA after 60 min or 5 min incubation has the same conformation, but the guide-target duplex has two different conformations (linear and kinked). Superposition is required to understand the differences between these two different conformations of RNA and DNA structures. Also, do nucleic acids of different complexes have similar linear conformations? Superposition is needed to compare the structures of guide-target duplexes and dsDNA.

4. In the 18-20 mm structure, has the TS been cleaved by the HNH domain? The HNH cleaves the TS, yielding products with 5'-phosphate and 3'-OH. The phosphate group contains four oxygen atoms instead of three. In addition, the density of 2-4 nt in the TS should be shown in the supplemental data.

5. In Fig. 2c, has the NTS been cleaved by the RuvC structural domain? Or is the NTS still intact, but other residues are disordered in the 18-20 mm structure? Density for two Mg2+ is required to show that two Mg2+ are bound in the RuvC catalytic domain.

Minor:

1. Ext. Data Fig. 4a: The authors found that the 6-8 mm and 9-11 mm DNA has poor binding affinity to Cas9 as the fully unmatched DNA. However, DNA binding of SpCas9 is mediated by PAM-proximal RNA:DNA base-pairing (Zhang, NAR, 2020). Additionally, another submitted paper showed that the presence of six complementary nt in the PAM-proximal region of the target DNA heteroduplex is sufficient for stable association with the SpCas9-guide RNA complex.

2. In Fig. 3b, Y1010 interacts with A2 of gRNA, but in Fig. 3c Y1010 interacts with C3 in the target strand and C18 in the sgRNA. In addition, the label of amino acids in Fig. 3c may be incorrect.


Referee #2:

Bravo, Liu and co-workers describe a study in which they aimed to reveal the molecular basis of mismatch base pairing by SpCas9. To do so, kinetic analysis has been used in combination with cryo-EM-based structural analyses of Cas9 at different stages of mismatch surveillance. In the case of PAM-distal mismatches, a linear conformation of the duplex appears to be formed between the guide RNA (gRNA) and DNA target strand (TS). It is concluded that the latter conformation prevents Cas9 activation, as a kinked gRNA:TS duplex is believed to be required for domain rearrangements that eventually allow for cleavage of both DNA strands. In case of 'highly tolerated PAM-distal mismatches', a kinked conformation of a distorted duplex conformation appears to be stabilized by a flexible loop of the RuvC domain. The authors conclude that the gained molecular insights may be used for future design of new high-fidelity/high-activity Cas9 variants. Impressive work, well executed. The presentation of the work in text and figures is good, but contains several minor mistakes that should be addressed (see below).

Major comments

To analyse the kinetics of the Cas9 nuclease activity, an assay was used with a FAM label at the 5' end of the target strand. HNH activity would result in cleavage of the target strand, that was detected. However, can the authors rule out that this (in part) represents nicking rather than generation of double-strand breaks, i.e. does the HNH activity in all tested mismatch cases coincide with RuvC activity? I could imagine that in the case of mismatches there may be a higher levels of nicking. This should be tested by also labelling the NTS.

Kinking of the TS:gRNA correlates with cleavage activity (Fig. 1). Whereas details are provided on the kinking/cleavage in the case of the 18-20 MM (distorted TS:gRNA duplex), much less detail is provided for the 12-14 MM: what are the consequences of the mismatches at the level of the TS:gRNA helix, does it also result in an alternative, distorted structure? The authors should mention why they could not provide a model in the latter case, and speculate about the molecular mechanism (inspired by the insights of 18-20 MM).

I agree with the suggestion of the authors that the gained structural insights may provide a basis for engineering towards a Cas9 variants with enhanced specificity. Although it would greatly strengthen the paper to include such engineering here, the authors should at least be more explicit in describing what Cas9 residues they would modify to avoid cleavage of the 'highly tolerated' off-targets.

What is missing in the current study is an on-target control, that would allow answering an important question: is the here revealed activation mechanism an alternative solution that allows cleavage of some highly tolerated mismatches, or is this a way to trap transient intermediates that also occur (but maybe with a shorter half-life) during on-target activation? The authors should at least discuss this.

Another obvious way to strengthen the current paper would be to perform additional cleavage kinetics analysis and cryo-EM analysis of previously established high-fidelity Cas9 variants (referred to in the manuscript), and compare that to the here presented wild-type Cas9.

It is important to include a clear model of the different step of Cas9 targeting. However, what is missing in Fig. 4 is an R-loop structure, with two DNA strands and the gRNA. Also more details should be added (e.g. HNH, RuvC, L1, L2, cleavage sites). In the discussion, next to the kinking of the TS:gRNA, also the kinking of the dsDNA (Jiang, 2016) should be addressed.

Minor comments

• Overall the manuscript text should be polished.

• Main section - TYPO - In order to understand the UNDERLYING molecular mechanisms UNDERLYING off-target recognition, ...

• Concluding paragraph - TYPO - ... employs a unique mechanism TO FOR stabilization of a highly distorted duplex conformation, ...

• Fig. 1a - Improve the visibility of the difference between linear DNA and kinked DNA of all tested mismatches, just as has been done in Fig. 2a.

• Fig. 1c legend - [typo/text missing] - The [......] between PAM-proximal TS:NTS duplex and PAM-distal gRNA:TS duplex for each of the 6 structures determined.

• Ext. Data Fig. 3c - Mention that this is a scheme 18-20 MM; there are errors in the scheme: (i) the gRNA 5-end should be re-drawn (as in Fig. 3b): A2-G1-C3 > G1-A2-C3, and (ii) U6 in the NTS-DNA does not seem right. In addition, sequences do not match completely with sequences in Ext. Data Table 1.


Referee #3:

A Summary of the key results

In their manuscript, Bravo, Liu et al. provide novel structural insights into the mechanism by which Cas9-crRNA complexes recognize and cleave DNA targets. First, kinetic measurements have been performed to determine the effect of three-in-a-row mismatches at several positions along the crRNA:TS duplex. These experiments show that for certain positions these mismatches have detrimental effects on the cleavage rate as well as the maximum turnover, while on other positions (12-14MM and 18-20MM) cleavage rate is reduced while maximum turnover is maintained (~80%). Using this information, Cas9 in complex with a crRNA guide and different DNA targets was subjected to structure determination using cryoEM. By varying incubation times prior to vitrification, Cas9-crRNA-DNA complexes could be trapped in different conformations before the structures were solved. These structures provide various insights that researchers were unable to uncover in previous Cas9 structures and are a major progress in the understanding of how Cas9 functions.

The solved Cas9 cryoEM structures reveal that the crRNA:TS duplex can assume two different conformations: one in which it is kinked (as observed in previously determined structures of cleavage-competent Cas9), and one previously unknown conformation in which the DNA is linear. Cas9 in complex with targets that are cleaved inefficiently results mostly in the ‘linear’ conformation, while Cas9 in complex with targets that are cleaved efficiently (12-14MM and 18-20MM) are found mostly in the ‘kinked’ conformation. In these ‘kinked’ conformations, the crRNA-TS duplex has docked with the REC3 domain. Furthermore, various previously unknown interactions of L1 and L2 regions (which are located directly upstream and downstream the HNH domain) with the crRNA-TS duplex are hypothesized to be critical for a structural transition to the active state that eventually results in target cleavage. Lastly, a structural basis is given for the ‘allowed’ 18-20MM mismatches. Although not visible in previously determined structures, it is shown that an unexpected conformation of the RuvC residues stabilizes the mismatched crRNA-TS residues, resulting in a conformation in which one of the crRNA nucleotides is ‘flipped out’ and another is inserted in between TS nucleotides.


B Originality and significance

While previous studies have shed light on various aspects of the mechanism by which Cas9 mediates DNA cleavage, this study reveals a novel conformation in which the DNA remains linear before it assumes the known ‘kinked’ formation. Furthermore, the structures reveal hitherto unknown details of crRNA:TS coordination by the L1, L2, and RuvC domains, which is suggested to mediate interactions that are essential for Cas9-mediated target cleavage. The manuscript is well written and contains many novel aspects that will be of interest for the broad scientific community that studies and/or makes use of Cas9, and of major importance for the scientific community that engineers high-fidelity Cas9 mutants.


C Data and methodology

The methodology used is valid to investigate the mechanism of Cas9. I have several comments regarding the validity of interpretation and the quality of presentation, which are listed below.


D Appropriate use of statistics and treatment of uncertainties

-


E: Conclusions: robustness, validity, reliability

While I think the conclusions drawn are mostly robust and reliable, I have a few comments, mostly textual, some experimental, that I think should be addressed before publication.


F-H: Suggested improvements

Major comments

- What is the structural basis for ‘allowing’ the 12-14MM mismatches to be cleaved relatively efficiently? Are these mismatches tolerated because they are not located in a region critical for interactions that facilitate cleavage?

- While I do not doubt the structures justify most conclusions drawn by the authors, the figures should allow readers to draw the same conclusions. In Fig. 1a the different conformation that the target DNA or crRNA:TS duplex assumes is very difficult to see. Especially in the 18-20MM kinked structure, in which the HNH domain is visible, it is almost impossible to see differences in the DNA conformations. A figure similar to Fig. 2a might be more suitable for this, and lines could be drawn over the figure to indicate the difference between the linear or kinked DNA conformations. Similarly, while most figures beautifully illustrate the important residues and nucleotides, it is not possible to see what residues are interacting with what base or backbone phosphate. This is also not clear in Ext. Data Fig. 3. A more detailed schematic figure should be provided, or interactions should be indicated in the figures with dashed lines (unless the resolution does not allow to do this confidently).

- The observation that the RuvC active site is in the catalytically-competent conformation, including divalent cations and water, is exciting. Can the authors give a (supplementary) figure with the cryoEM map to corroborate the confidence of this conformation? Furthermore, can the authors propose a cleavage mechanism? Can the authors discuss the similarities and differences with other RuvC catalytic sites, for example the one proposed for Thermus thermophilus Argonaute (https://www.nature.com/articles/nature08434)? Furthermore, in the Casalino et al. paper in which MD simulations were used to predict the cleavage mechanism, two potential mechanisms are given. Does the observed conformation corroborate one or the other, and/or exclude the other mechanism?

- The authors indicate the arrangement of the HNH domain is ‘entirely consistent with the previously observed active Cas9 conformation’. However, the L1 and L2 domains are ‘often missing’ according to the authors. First, what is the explanation for the observed differences underlying the observation that the L1 linker docks against the crRNA:TS duplex observed in the structures reported earlier and the structures reported here? Does it suggest the positioning of these domains is flexible and perhaps not essential? Second, ‘often’ suggests that they are observed in certain cases. If so, have they not been identified or has their importance been overlooked?

- Given their importance, a detailed schematic representation should be given in which the interactions between L1 helix and L2 helix residues and the crRNA:TS duplex are made clear (e.g. similar to Fig. 3b). Or does the local resolution not allow to confidently map such interactions? It would be helpful if interactions could be given in a way that makes it clear with what the residues exactly interact (in fact, this is true for Fig. 3b, too). A schematic of interactions as displayed in this paper (https://www.nature.com/articles/nature13579, Fig. 2b), in which it is clear if residues interact with backbone phosphates or bases, would be helpful, if the resolution allows this. The map observed in Fig. 2d suggests it is possible to do this, but based on Ext. Data Fig. 3e might this be difficult? Is there also density observed for the waters indicated in Fig. 2d? This is not entirely clear from the figure.

- The authors claim interactions between the L1 linker and the crRNA:TS are a prerequisite for Cas9 activation and cleavage of the TS. The authors should make mutants for the L1 residues that facilitate these interactions to corroborate that theory. Similarly, a residue F916 mutant should be made to corroborate the suggested importance of F916 for RuvC-mediated cleavage of the NTS. Such mutants should be investigated for their ability to cleave the TS and NTS, to prove the theory made based on the structural observations.

Minor and textual comments

Page 2:
- The general convention is to use CRISPR-Cas, not CRISPR/Cas.

- Reduced cleavage is observed for the 6-8MM, 9-11MM, and 15-17MM DNA targets (up to maximum 20%; Ext. Data Fig. 1). Can the authors discuss why it plateaus at this percentage? Furthermore, clearly cleavage is not completely inhibited. Can the authors explain why no kinked DNA conformations are observed for the 15-17MM targets after 60 min?

- Can the authors discuss if the mismatches block (active) the kinked formation, or do they prevent (passive) formation of the kinked formation because the crRNA:TS duplex cannot properly form?

- The authors compare the cleavage rate of 12-14MM to 18-20MM (10-fold change). Isn’t it more logical to compare the cleavage rate to Cas9 incubated with fully matched targets?

- The authors indicate a ‘well-characterized PAM-distal 18-20MM” --> they should cite the papers in which this mismatch is characterized.

- “much slower” is used to explain the cleavage rates for 6-8MM and 9-11MM. It would be more informative to give a fold-change.

-...the endonuclease HNH... --> ...the endonuclease domain HNH...

Page 3:
- ...that mismatches that prevent PAM-distal... --> ...that mismatches that prevent the PAM-distal.... Also this hypothesis is not completely clear to me at this point. Perhaps it can be rephrased as “we hypothesized that mismatches that prevent the PAM-distal gRNA-TS duplex from docking on REC3 will be unable to assume the kinked conformation and consequently would have severely reduced DNA cleavage” (if, at least, that is what the authors mean).

- The paragraph title “18-20 mismatch induces a fully active Cas9 conformation” suggests that the mismatch itself induces the conformation, which (I assume) is not what the authors mean.

- I did not completely understand the numbering of the crRNA/TS/NTS in the figures until I saw Ext. Data Fig. 3. It would be helpful to add a figure panel in one of the main figures in which the sequences and numbering is indicated.

Page 4:
- Experiments can corroborate the theory that REC3 binding to the crRNA:TS duplex is important for target cleavage. To corroborate this theory, the authors refer to previously made high-fidelity mutant Cas9 enzymes, which is justified. However, a figure should be given in which REC3 residues interacting with the crRNA:TS are displayed. Furthermore, in the Fig. 4 text, it is stated that ‘REC3 mutants responsible for PAM-distal end docking destabilize the kinked conformation and therefore prevent L1 docking and Cas9 activation’. Do the authors refer to their own work here or to the high-fidelity mutants?

Ext. Data Fig. 1:
- What does A indicate? Total cleavage?

Ext. Data Fig. 4:
- How is the apparent binding affinity determined? This is not clear from the methods. Why is a normalized value given, and not an absolute value?

Table 3:
- I think at this resolution no Ramachandran outliers should be allowed unless they are justified. Can the Ramachandran outliers be explained?

**Author Rebuttals to Initial Comments:**

Referee #1:

CRISPR/Cas9 as a programmable genome editing tool has been hindered by off-target DNA cleavage. Bravo et al. performed enzyme kinetics to investigate the impact of mismatches within different regions on SpCas9 domain rearrangement during R-loop extension. The authors determined a series of structures with mismatches at different positions, including 12-14 mm, 15-17 mm and 18-20 mm. These structures provide snapshots of the conformation of SpCas9 bound to the mismatched target DNA. The authors also captured the RuvC catalytically-competent conformation with the non-target strand bound in the active site. Together, this structural study provides new conformational change of Cas9 for the activation, and may provide hints for the rational design of Super Cas9 with higher fidelity, i.e., disrupting conformations that bind to mismatched targets while avoiding disrupting interactions to stabilize intermediate states that bind to the correct target DNA.

Major:

1. In the 18-20 mm structure, the loop of residues 1009-1016 in the RuvC domain interacts with the mismatch residues to stabilize the unusual nucleic acid conformation. The authors found that these interactions were only present in the 18-20 mm complex and not in the target complex. How do mutations in these residues that interact with mismatched nucleotides affect the cleavage of mismatched targets and target DNA by Cas9, respectively? Do these amino acid mutations increase the accuracy of Cas9?

This is an excellent point. In the revised manuscript, we now include data that shows that this loop does have an effect on cleavage of DNA. We mutated all 7 of the RuvC loop residues, which interact with the mismatched nucleotides, to aspartates (7-D Cas9). We observed that this mutant cleaves on-target DNA at a nearly identical rate as wild-type Cas9 (2 s^-1^ vs 1 s^-1^) but cleaves 18-20 MM off-target DNA 500-fold slower than on-target. We subsequently performed a cleavage competition assay, where on- and off-target (18-20 MM) dsDNA substrates are simultaneously provided to WT Cas9 or 7-D Cas9. We observed that WT Cas9 has a 1.55-fold discrimination between on- and off-target substrates, whereas 7-D Cas9 has a 6.3-fold discrimination. Since 7-D Cas9 has improved fidelity for DNA cleavage without a compromised on-target cleavage rate, we have chosen to refer to this mutant as SuperFi-Cas9. We have now added this data as Figure 4e-g, and added the following text:

“To validate the role of this loop for mismatch stabilization, we designed a 7-D mutant (where all seven of the stabilizing residues in Fig. 4b are mutated to aspartate) and tested the effects of this mutant on DNA cleavage. While this 7-D mutant cleaved on-target DNA with a similar rate to WT SpCas9 (2 s^-1^), we observed that cleavage of 18-20 MM DNA was 500-fold slower (0.004 s^-1^) (**Fig 4e**). This indicates that this loop is critical for stabilizing the distorted mismatch-induced PAM-distal duplex conformation, thereby allowing the duplex to adopt the kinked conformation that is prerequisite for Cas9 activation. We have named this designed high-fidelity variant that retains wild type on-target cleavage rate, SuperFi-Cas9.

To test the change in specificity, we performed a direct competition assay, where on-target and 18-20 MM dsDNA substrates were mixed simultaneously with either enzyme and cleavage was monitored over time. While WT Cas9 showed some preference for on-target substrates (a 1.55-fold ratio between the on-target and 18-20 MM off-target product formed), SuperFi-Cas9 showed rapid cleavage of on-target while showing minimal cleavage of 18-20 MM DNA (6.3-fold preference for on-target DNA (**Fig 4f & g**). The unique ability to discriminate between on- and off-target DNA substrates without compromising DNA cleavage efficiency appears to be unique to SuperFi-Cas9 (Kim et al., 2020). While further studies are needed to fully define the kinetic basis for the change in discrimination, our current data provide a proof of concept and provide a rationale for engineering improved variants of Cas9 using our structure.”

2. It is easy to understand that the mismatches at the PAM distal region have less effect than at other positions along the sgRNA-TS duplex. Why does 15-17 mm have lower activity than 12-14 mm? What is the detailed conformation of the mismatched 12-14 and 15-17 base pairs?

Again, this is a good question. Analysis of our structures reveals that due to the phase of the gRNA:TS duplex, positions 12-14 are unique in that they make no contacts with the REC lobe of Cas9. Positions 9-11 and 15-17 make extensive contacts, particularly with the REC3 domain. Since REC3 has been demonstrated to act as a critical determinant of whether PAM-distal mismatches are “sensed” by Cas9, our structural analysis demonstrates that positions 12-14 are a ‘blind spot’ with REC3 being less capable of discriminating on- vs off-targets at these positions than at adjacent ones.

We have incorporated a new main figure (**Fig 2**) to demonstrate this, and added the following text:

“Interestingly, positions 12-14 of the gRNA:TS make no direct contacts with the REC3 domain of Cas9 (**Fig 2**). While positions 9-11 and 15-17 make significant contacts with REC3, the alignment of the gRNA:TS duplex leaves positions 12-14 without any engagement with this domain (**Fig 2d,e**). Since REC3 plays a critical role in sensing PAM-distal mismatches (Chen et al., 2017), the 12-14 MM is likely able to evade mismatch discrimination by REC3 since it is positioned in a blind-spot.”

3. The Cas9 in the complex of Cas9-12-14 mm DNA after 60 min or 5 min incubation has the same conformation, but the guide-target duplex has two different conformations (linear and kinked). Superposition is required to understand the differences between these two different conformations of RNA and DNA structures. Also, do nucleic acids of different complexes have similar linear conformations? Superposition is needed to compare the structures of guide-target duplexes and dsDNA.

In the revised manuscript, **Fig 2** shows the two conformations of the 12-14MM complexes along side of one another. We have also added a citation for the preprint from the Jinek group (Pacesa et al., 2021), which suggests that linear gRNA:TS duplex conformations are present as intermediates of R-loop formation. We have added the following text:

“This is supported by recent structural analyses of catalytically dead Cas9 in complex with various R-loop formation intermediates, several of which exhibit linear gRNA:TS duplex conformations that are similar to our linear duplex structures (Pacesa and Jinek, 2021).”

4. In the 18-20 mm structure, has the TS been cleaved by the HNH domain? The HNH cleaves the TS, yielding products with 5'-phosphate and 3'-OH. The phosphate group contains four oxygen atoms instead of three. In addition, the density of 2-4 nt in the TS should be shown in the supplemental data.

Indeed, the TS has been cleaved in the 18-20 MM kinked structure. We have revised our model accordingly so that the 5’ phosphate has four oxygens. We have also added Extended Data Fig 3, which shows the cryo-EM density of this active site. It also shows the cryo-EM density for the RuvC active site, L1 and L2 linkers, respectively.

5. In Fig. 2c, has the NTS been cleaved by the RuvC structural domain? Or is the NTS still intact, but other residues are disordered in the 18-20 mm structure? Density for two Mg2+ is required to show that two Mg2+ are bound in the RuvC catalytic domain.

We apologize for the confusion. We have now edited Fig 2 to improve the clarity, showing that the NTS has indeed been cleaved. We have also added a new Extended Data Figure 3, showing the cryo-EM density for the RuvC active site.

Minor:

1. Ext. Data Fig. 4a: The authors found that the 6-8 mm and 9-11 mm DNA has poor binding affinity to Cas9 as the fully unmatched DNA. However, DNA binding of SpCas9 is mediated by PAM-proximal RNA:DNA base-pairing (Zhang, NAR, 2020). Additionally, another submitted paper showed that the presence of six complementary nt in the PAM-proximal region of the target DNA heteroduplex is sufficient for stable association with the SpCas9-guide RNA complex.

We thank the reviewer for their insight. Since this data has already been published elsewhere (Jones et al., Nat Biotech 2021), and multiple reviewers have questioned the presentation of this data, we have decided to remove it from our manuscript. We initially included the data to support our claim that 15-17MM substrate was able to form an R-loop, but considering the new data from the Jinek group, we now feel that this data is no longer necessary to include. We have cited the Jinek group’s preprint instead.

2. In Fig. 3b, Y1010 interacts with A2 of gRNA, but in Fig. 3c Y1010 interacts with C3 in the target strand and C18 in the sgRNA. In addition, the label of amino acids in Fig. 3c may be incorrect.

We thank the reviewer for pointing out this oversight. This has been corrected in the revised manuscript.

Referee #2:

Bravo, Liu and co-workers describe a study in which they aimed to reveal the molecular basis of mismatch base pairing by SpCas9. To do so, kinetic analysis has been used in combination with cryo-EM-based structural analyses of Cas9 at different stages of mismatch surveillance. In the case of PAM-distal mismatches, a linear conformation of the duplex appears to be formed between the guide RNA (gRNA) and DNA target strand (TS). It is concluded that the latter conformation prevents Cas9 activation, as a kinked gRNA:TS duplex is believed to be required for domain rearrangements that eventually allow for cleavage of both DNA strands. In case of 'highly tolerated PAM-distal mismatches', a kinked conformation of a distorted duplex conformation appears to be stabilized by a flexible loop of the RuvC domain. The authors conclude that the gained molecular insights may be used for future design of new high-fidelity/high-activity Cas9 variants. Impressive work, well executed. The
presentation of the work in text and figures is good, but contains several minor mistakes that should be addressed (see below).

Major comments
To analyse the kinetics of the Cas9 nuclease activity, an assay was used with a FAM label at the 5' end of the target strand. HNH activity would result in cleavage of the target strand, that was detected. However, can the authors rule out that this (in part) represents nicking rather than generation of double-strand breaks, i.e. does the HNH activity in all tested mismatch cases coincide with RuvC activity? I could imagine that in the case of mismatches there may be a higher levels of nicking. This should be tested by also labelling the NTS.

In our previous work (Liu et al., Nat Comms, 2020 and Gong et al., Cell Rep, 2018), we have determined the rates of non-target strand cleavage for Cas9 on on-target and 18-20 MM substrates. We now provide NTS cleavage data for the 12-14 MM in Extended Data figure 1. The 12-14 MM NTS is cleaved by Cas9 at a slower rate than the TS, as is expected.

Kinking of the TS:gRNA correlates with cleavage activity (Fig. 1). Whereas details are provided on the kinking/cleavage in the case of the 18-20 MM (distorted TS:gRNA duplex), much less detail is provided for the 12-14 MM: what are the consequences of the mismatches at the level of the TS:gRNA helix, does it also result in an alternative, distorted structure? The authors should mention why they could not provide a model in the latter case, and speculate about the molecular mechanism (inspired by the insights of 18-20 MM).

We have revised Figure 1 to explicitly show the conformation of the gRNA:TS duplex observed in our structures. We have also added a new Figure 2 that provides further insights into why the 12-14MM is able to cryptically activate Cas9. This is described in detail above in response to Reviewer #1.

I agree with the suggestion of the authors that the gained structural insights may provide a basis for engineering towards a Cas9 variants with enhanced specificity. Although it would greatly strengthen the paper to include such engineering here, the authors should at least be more explicit in describing what Cas9 residues they would modify to avoid cleavage of the 'highly tolerated' off-targets.

Our new high fidelity variant SuperFi Cas9 has been tested and the mutations listet. This has been addressed above in the response to Reviewer 1.

What is missing in the current study is an on-target control, that would allow answering an important question: is the here revealed activation mechanism an alternative solution that allows cleavage of some highly tolerated mismatches, or is this a way to trap transient intermediates that also occur (but maybe with a shorter half-life) during on-target activation? The authors should at least discuss this.

We appreciate the concerns of the reviewer. Since there have been many structures of on-target Cas9 complexes determined to date, we have added a new Extended Data Figure 5 that provides detailed comparisons of the RuvC loop identified in our 18-20 MM product state structure. In both crystallographic and cryo-EM maps, the RuvC loop is absent in 5 different on-target structures (4UN3, 5F9R, 5OO8, 6O0Y, 6O0X). Extended Data Figure 5 shows experimental electron and cryo-EM densities (or lack thereof) for this region. Based on this analysis, we are confident that the mechanism of PAM-distal mismatch tolerance we have discovered is specific for mismatches.

While our manuscript was in preparation, two other groups have determined structures of Cas9 R-loop structures that the authors believe represent intermediates, either through chemical crosslinking or trapping catalytically inactive Cas9 with DNA targets with various complementarities to the gRNA. Both papers are cited in our revised manuscript (Pacesa et al., 2021, Cofsky et al., 2021). However, for a species to be deemed an intermediate, it must pass the test for kinetic competence (Anderson and Johnson, J. Biol. Chem. 1990, 265:5567-5572), which is not possible using crosslinks or dead enzyme.

Another obvious way to strengthen the current paper would be to perform additional cleavage kinetics analysis and cryo-EM analysis of previously established high-fidelity Cas9 variants (referred to in the manuscript), and compare that to the here presented wild-type Cas9.

We have already published a manuscript describing in-depth kinetic analysis of high fidelity Cas9 variants (HF-1 and Hypa) in the presence of the 18-20MM (Liu et al., 2020). Structural analysis of these variants is beyond the scope of this paper.

It is important to include a clear model of the different step of Cas9 targeting. However, what is missing in Fig. 4 is an R-loop structure, with two DNA strands and the gRNA. Also more details should be added (e.g. HNH, RuvC, L1, L2, cleavage sites). In the discussion, next to the kinking of the TS:gRNA, also the kinking of the dsDNA (Jiang, 2016) should be addressed.

This is a great suggestion. We have revised our schematic figure (now Figure 5) to delineate R-loop formation from R-loop completion and PAM-distal duplex kinking. We have also added labels for the TS, NTS, gRNA, L1, L2, HNH and RuvC to the schematic to improve visual clarity. The legend for Figure 5 now reads as follows:

“**Fig. 5 | Model for Cas9 activation.** During R-loop propagation (step 1), the gRNA:TS duplex adopts a linear conformation. After R-loop completion, the PAM-distal end of the linear duplex is captured by REC3 (steps 2 & 3). Mismatches in the PAM-distal region appear to prevent REC3 docking and thereby block subsequent steps of Cas9 activation. Once the kinked R-loop conformation has been formed, L1 and L2 linkers utilize the gRNA:TS duplex as a scaffold to position the HNH domain at the scissile phosphate of the TS and to position the NTS in the RuvC site (step 4), enabling Cas9 to make a double-strand break (step 5). According to this model, mutations in the RuvC loop (corresponding to SuperFi-Cas9) inhibit formation of the kinked conformation and subsequent cleavage of gRNA:TS duplex with mismatches at the PAM-distal end.”

The corresponding author (Taylor) described the DNA as kinked in his cryo-EM structure presented in Jiang, 2016. The DNA duplex conformation is a direct consequence of the gRNA:TS kinking since the dsDNA is present immediately adjacent to the R-loop. If the R-loop is kinked, the dsDNA either side of it will be kinked, so these are identical steps.

Minor comments

• Overall the manuscript text should be polished.

• Main section - TYPO - In order to understand the UNDERLYING molecular mechanisms UNDERLYING off-target recognition, ...

Done.

• Concluding paragraph - TYPO - ... employs a unique mechanism TO FOR stabilization of a highly distorted duplex conformation, ...

Done.

• Fig. 1a - Improve the visibility of the difference between linear DNA and kinked DNA of all tested mismatches, just as has been done in Fig. 2a.

Done.

• Fig. 1c legend - [typo/text missing] - The [......] between PAM-proximal TS:NTS duplex and PAM-distal gRNA:TS duplex for each of the 6 structures determined.

Done.

• Ext. Data Fig. 3c - Mention that this is a scheme 18-20 MM; there are errors in the scheme: (i) the gRNA 5-end should be re-drawn (as in Fig. 3b): A2-G1-C3 > G1-A2-C3, and (ii) U6 in the NTS-DNA does not seem right. In addition, sequences do not match completely with sequences in Ext. Data Table 1.

This figure has been updated. The sequences depicted correspond to the middle of the duplex, since the extreme end of the TS:NTS duplex are poorly resolved in our maps and have little to do with our conclusions. To make data interpretation easier, we have renamed and renumbered our sequences in our figures based on the position of the TS:gRNA duplex.


Referee #3:

A Summary of the key results

In their manuscript, Bravo, Liu et al. provide novel structural insights into the mechanism by which Cas9-crRNA complexes recognize and cleave DNA targets. First, kinetic measurements have been performed to determine the effect of three-in-a-row mismatches at several positions along the crRNA:TS duplex. These experiments show that for certain positions these mismatches have detrimental effects on the cleavage rate as well as the maximum turnover, while on other positions (12-14MM and 18-20MM) cleavage rate is reduced while maximum turnover is maintained (~80%). Using this information, Cas9 in complex with a crRNA guide and different DNA targets was subjected to structure determination using cryoEM. By varying incubation times prior to vitrification, Cas9-crRNA-DNA complexes could be trapped in different conformations before the structures were solved. These structures provide various insights that researchers were unable to uncover in previous Cas9 structures and are a major progress in the understanding of how Cas9 functions.

The solved Cas9 cryoEM structures reveal that the crRNA:TS duplex can assume two different conformations: one in which it is kinked (as observed in previously determined structures of cleavage-competent Cas9), and one previously unknown conformation in which the DNA is linear. Cas9 in complex with targets that are cleaved inefficiently results mostly in the ‘linear’ conformation, while Cas9 in complex with targets that are cleaved efficiently (12-14MM and 18-20MM) are found mostly in the ‘kinked’ conformation. In these ‘kinked’ conformations, the crRNA-TS duplex has docked with the REC3 domain. Furthermore, various previously unknown interactions of L1 and L2 regions (which are located directly upstream and downstream the HNH domain) with the crRNA-TS duplex are hypothesized to be critical for a structural transition to the active state that eventually results in target cleavage. Lastly, a structural basis is given for the ‘allowed’ 18-20MM mismatches. Although not visible in previously determined structures, it is shown that an unexpected conformation of the RuvC residues stabilizes the mismatched crRNA-TS residues, resulting in a conformation in which one of the crRNA nucleotides is ‘flipped out’ and another is inserted in between TS nucleotides.


B Originality and significance

While previous studies have shed light on various aspects of the mechanism by which Cas9 mediates DNA cleavage, this study reveals a novel conformation in which the DNA remains linear before it assumes the known ‘kinked’ formation. Furthermore, the structures reveal hitherto unknown details of crRNA:TS coordination by the L1, L2, and RuvC domains, which is suggested to mediate interactions that are essential for Cas9-mediated target cleavage. The manuscript is well written and contains many novel aspects that will be of interest for the broad scientific community that studies and/or makes use of Cas9, and of major importance for the scientific community that engineers high-fidelity Cas9 mutants.


C Data and methodology

The methodology used is valid to investigate the mechanism of Cas9. I have several comments regarding the validity of interpretation and the quality of presentation, which are listed below.


D Appropriate use of statistics and treatment of uncertainties

-


E: Conclusions: robustness, validity, reliability

While I think the conclusions drawn are mostly robust and reliable, I have a few comments, mostly textual, some experimental, that I think should be addressed before publication.


F-H: Suggested improvements 

Major comments

- What is the structural basis for ‘allowing’ the 12-14MM mismatches to be cleaved relatively efficiently? Are these mismatches tolerated because they are not located in a region critical for interactions that facilitate cleavage?

This is a good question. We have provided a detailed answer in our responses to Reviewer 1 and Reviewer 2.

- While I do not doubt the structures justify most conclusions drawn by the authors, the figures should allow readers to draw the same conclusions. In Fig. 1a the different conformation that the target DNA or crRNA:TS duplex assumes is very difficult to see. Especially in the 18-20MM kinked structure, in which the HNH domain is visible, it is almost impossible to see differences in the DNA conformations. A figure similar to Fig. 2a might be more suitable for this, and lines could be drawn over the figure to indicate the difference between the linear or kinked DNA conformations. Similarly, while most figures beautifully illustrate the important residues and nucleotides, it is not possible to see what residues are interacting with what base or backbone phosphate. This is also not clear in Ext. Data Fig. 3. A more detailed schematic figure should be provided, or interactions should be indicated in the figures with dashed lines (unless the resolution does not allow to do this
confidently).

We appreciate the reviewer’s concern. A new panel has been added to Figure 1 to show the angle between the PAM-proximal and -distal ends of the gRNA:TS duplex. We feel that adding lines to Extended Data Figure 3 makes visualizing interactions harder since there are so many interactions that the figure becomes impossible to interpret. Interactions involving water molecules or Mg2+ ions have been denoted by dashed lines. We have now added more labels to indicate the various domains from which the interacting residues originate to improve clarity.

- The observation that the RuvC active site is in the catalytically-competent conformation, including divalent cations and water, is exciting. Can the authors give a (supplementary) figure with the cryoEM map to corroborate the confidence of this conformation? Furthermore, can the authors propose a cleavage mechanism? Can the authors discuss the similarities and differences with other RuvC catalytic sites, for example the one proposed for Thermus thermophilus Argonaute (<https://www.nature.com/articles/nature08434>)? Furthermore, in the Casalino et al. paper in which MD simulations were used to predict the cleavage mechanism, two potential mechanisms are given. Does the observed conformation corroborate one or the other, and/or exclude the other mechanism?

We agree with the reviewer that the bona fide product state of the RuvC site is very exciting. Based on our structure, we can say that catalysis occurs via a H893-mediated pathway, rather than the alternative phosphate-mediated pathway, which were the two potential mechanisms proposed in the Casalino et al. paper. The histidine-mediated pathway has a distance between the two catalytic Mg2+ ions of 4.2 Å (in very good agreement with our structure), whereas the phosphate-mediated pathway has an alternative distance of 4.9 Å. Our results follow the Steitz & Steitz two-metal-ion dependent mechanism.

We have added a new Extended Data Figure 3 with detailed views of cryo-EM density for the HNH active site, RuvC active site, and L1 linker. We have also added a putative chemical mechanism for the RuvC site and added the following to the main body of text:

“Our structure suggests a histidine-mediated catalytic mechanism, consistent with two-metal-ion dependent catalysis (Steitz and Steitz, 1993) and supported by quantum-classical simulations (Casalino et al., 2020). Furthermore, our product state reveals that the two Mg^2+^ ions have a distance of ~4.2Å from each other, in agreement with the product state of the histidine-mediated mechanism (**Extended Data Fig. 3**).”

- The authors indicate the arrangement of the HNH domain is ‘entirely consistent with the previously observed active Cas9 conformation’. However, the L1 and L2 domains are ‘often missing’ according to the authors. First, what is the explanation for the observed differences underlying the observation that the L1 linker docks against the crRNA:TS duplex observed in the structures reported earlier and the structures reported here? Does it suggest the positioning of these domains is flexible and perhaps not essential? Second, ‘often’ suggests that they are observed in certain cases. If so, have they not been identified or has their importance been overlooked?

We apologize for the confusion. In inactive Cas9 structures (e.g. 4UN3, 5F9R or 4OO8) where HNH is positioned away from the TS cleavage site, the L2 linker is frequently disordered. In the active Cas9 structures captured by cryo-EM in Zhu et al., 2019 the TS has been cleaved, and the L1 linker helix has well-resolved density (albeit not of suitable resolution to visualize interactions between side-chains and the gRNA:TS duplex). This is depicted in Extended Data Figure 6 panel E where a cryo-EM map of Cas9 in an inactive conformation (EMD-3276 from Jiang et al., 2016) and an active conformation (EMD-0584) are compared.

Furthermore, active structures of StCas9 and NmeCas9 (class II-A and II-C CRISPR systems, respectively) have L1 linker helices that utilize the kinked PAM-distal gRNA:TS duplex to position HNH at the TS scissile phosphate. In summary, in all existing structures of Cas9 determined to date where HNH is positioned so that it can cleave the TS, L1 has been rearranged and docks upon the gRNA:TS duplex. However, in all structures of Cas9 that are incompatible with HNH cleavage of TS, the L1 helix is not docked on the gRNA:TS duplex. We feel that this structural analysis explains how HNH can undergo a 140º rotation to stably reposition to cleave the TS. The positioning of these L1 and L2 domains is indeed flexible but appears to be a prerequisite for cleavage.

We have added Extended Data Figure 7 with alignments of our active product structure and previously published structures of Cas9.

- Given their importance, a detailed schematic representation should be given in which the interactions between L1 helix and L2 helix residues and the crRNA:TS duplex are made clear (e.g. similar to Fig. 3b). Or does the local resolution not allow to confidently map such interactions? It would be helpful if interactions could be given in a way that makes it clear with what the residues exactly interact (in fact, this is true for Fig. 3b, too). A schematic of interactions as displayed in this paper (<https://www.nature.com/articles/nature13579>, Fig. 2b), in which it is clear if residues interact with backbone phosphates or bases, would be helpful, if the resolution allows this. The map observed in Fig. 2d suggests it is possible to do this, but based on Ext. Data Fig. 3e might this be difficult? Is there also density observed for the waters indicated in Fig. 2d? This is not entirely clear from the figure.

The local resolution of L1 is ~2.5Å and is sufficient to visualize such interactions. Figure 2 shows an isosurface representation rather than cryo-EM density. Extended Data Figure 3 has been added to show the cryo-EM density of L1 interactions with the gRNA:TS duplex. This has been clarified in the figure legend.

- The authors claim interactions between the L1 linker and the crRNA:TS are a prerequisite for Cas9 activation and cleavage of the TS. The authors should make mutants for the L1 residues that facilitate these interactions to corroborate that theory. Similarly, a residue F916 mutant should be made to corroborate the suggested importance of F916 for RuvC-mediated cleavage of the NTS. Such mutants should be investigated for their ability to cleave the TS and NTS, to prove the theory made based on the structural observations.

F916 positioning the NTS in the RuvC active site has been established in previous work (Jiang et al., 2016). In the StCas9 structure, a F673 within L2 was observed to play a similar role as SpCas9 F916. The authors of this study also found that a F673A mutation severely reduced but did not abolish DNA cleavage.

Our thorough structural analysis of all available Cas9 structures (including but not limited to: the 5 structures depicted in Extended Data Figure 5, the 6 inactive R-loop intermediate structures in the Pacesa & Jinek preprint, the 6 structures presented in our work, structures of other type II-A, II-B and II-C Cas9), the roles of L1 and L2 have been firmly established.

Minor and textual comments

Page 2:
- The general convention is to use CRISPR-Cas, not CRISPR/Cas.

This has been changed throughout the manuscript.

- Reduced cleavage is observed for the 6-8MM, 9-11MM, and 15-17MM DNA targets (up to maximum 20%; Ext. Data Fig. 1). Can the authors discuss why it plateaus at this percentage? Furthermore, clearly cleavage is not completely inhibited. Can the authors explain why no kinked DNA conformations are observed for the 15-17MM targets after 60 min?

We have pursued further 2D classification of this dataset and can visualize a mixture of linear and kinked conformations (see below). The population is clearly predominately linear. Due to this heterogeneity, it was not possible to determine a 3D reconstruction of a kinked conformation. We hypothesize that a very small fraction of the 15-17MM can adopt the kinked conformation after an hour of incubation, hence the ~20% of product formed. However, the presence of this particular mismatch strongly favors the linear conformation and cleavage is four orders of magnitude slower than on-target cleavage.

- Can the authors discuss if the mismatches block (active) the kinked formation, or do they prevent (passive) formation of the kinked formation because the crRNA:TS duplex cannot properly form?

It is likely the latter; however, we have no other supporting data to make a solid conclusion. We have therefore decided not to speculate on these models in our manuscript.

- The authors compare the cleavage rate of 12-14MM to 18-20MM (10-fold change). Isn’t it more logical to compare the cleavage rate to Cas9 incubated with fully matched targets?

This has been changed throughout the manuscript.

- The authors indicate a ‘well-characterized PAM-distal 18-20MM” --> they should cite the papers in which this mismatch is characterized.
This has been changed throughout the manuscript.

- “much slower” is used to explain the cleavage rates for 6-8MM and 9-11MM. It would be more informative to give a fold-change.
This has been changed throughout the manuscript.

-...the endonuclease HNH... --> ...the endonuclease domain HNH...

This has been changed throughout the manuscript.

Page 3:
- ...that mismatches that prevent PAM-distal... --> ...that mismatches that prevent the PAM-distal.... Also this hypothesis is not completely clear to me at this point. Perhaps it can be rephrased as “we hypothesized that mismatches that prevent the PAM-distal gRNA-TS duplex from docking on REC3 will be unable to assume the kinked conformation and consequently would have severely reduced DNA cleavage” (if, at least, that is what the authors mean).

We thank the reviewer for pointing this out. This has been changed as suggested.

- The paragraph title “18-20 mismatch induces a fully active Cas9 conformation” suggests that the mismatch itself induces the conformation, which (I assume) is not what the authors mean.

We have changed this as follows:

“18-20 mismatch supports Cas9 conformational activation”.

- I did not completely understand the numbering of the crRNA/TS/NTS in the figures until I saw Ext. Data Fig. 3. It would be helpful to add a figure panel in one of the main figures in which the sequences and numbering is indicated.

In the new Figure 2, the numbering is shown in the schematic figure and is described in the figure legend as follows:

“Numbering of the duplex starts with position 1 as the first TS base that hybridizes with the gRNA spacer.”

Page 4:
- Experiments can corroborate the theory that REC3 binding to the crRNA:TS duplex is important for target cleavage. To corroborate this theory, the authors refer to previously made high-fidelity mutant Cas9 enzymes, which is justified. However, a figure should be given in which REC3 residues interacting with the crRNA:TS are displayed. Furthermore, in the Fig. 4 text, it is stated that ‘REC3 mutants responsible for PAM-distal end docking destabilize the kinked conformation and therefore prevent L1 docking and Cas9 activation’. Do the authors refer to their own work here or to the high-fidelity mutants?

Ext. Data Fig. 1:
- What does A indicate? Total cleavage?

Yes. This has been amended in the figure legend for clarity as follows:

“*A*_obs_ corresponds to amplitude of product formed i.e. total cleavage.”

Ext. Data Fig. 4: 
- How is the apparent binding affinity determined? This is not clear from the methods. Why is a normalized value given, and not an absolute value?

This data analyses was performed in Jones et al., whereby a multiplexed high-throughput measurements of Cas9 binding to various DNA sequence was measured at a range of protein concentrations, giving a proxy for binding affinity. Since this data has already been published, we believe that this reanalysis is unnecessary for our story and have now removed this figure.

Table 3: 
- I think at this resolution no Ramachandran outliers should be allowed unless they are justified. Can the Ramachandran outliers be explained?

We have further refined our structures, and there are no longer any Ramachandran outliers.

**Reviewer Reports on the First Revision:**

Referee #1:

The revised manuscript has been improved by the inclusion of new data--in particular, the cleavage result with the 7-aa mutant of the RuvC loop, which interacts with the mismatched nucleotides. The cleavage of 18-20 MM of DNA is greatly reduced, while there is no effect on the cleavage of the target DNA. This new data paves the way for an improved variant of Cas9 that is able to describe both targeted and non-targeted situations in the distal region of the PAM. I still have some minor concerns.

1. In the Cas9-12-14 MM DNA complex, Cas9 is similar in the linear and the kinked conformation, but the gRNA:TS duplex is different. The authors aligned these two structures in Fig. 2c, but this figure fails to clearly show the difference and similarity between the linear and the kinked structures. An additional Extended Data figure to superimpose the nucleic acids in these two complexes without protein may show the differences of the linear and kinked conformations.
 
2. In the Cas9-12-14 MM DNA complex, a linear and a kinked duplex conformation are resolved. However, the authors mention in line 79 that "Cas9 has the same conformation in the two kinked duplex structures (Figure 2)." What do these two kinked conformations refer to?

3. The authors observed a product binding state for the RuvC domain, with nucleotides C18 and G19 positioned in its catalytic pocket. What about the other product containing nucleotides 1-17? The ssDNA substrate binding state with two Mg2+ of the RuvC domain of Cas12i2 were reported last year. Comparison of the substrate and product binding states of the RuvC domain may provide additional insight into the molecular mechanism of the RuvC domain.

Referee #2:

The manuscript has been substantially improved, all concerns that I have raised were appropriately addressed - well done!!


Referee #3:

I would like to thank the authors for addressing my comments appropriately.

I have some remaining minor remarks:

- The figure text of Fig. 1 panel says (b), but I think the text after it still belongs to panel (a).

- Panel labels in the (Ext. Data) figure text are sometimes capitalized, sometimes not.

- I appreciate that in Fig. 3d it is indicated that the 'map' is an isosurface presentation. However, for other figures also showing a 'map/density/surface' around the atoms, it should be stated in the figure text whether this is a cryoEM density map or an isosurface representation, and as for Fig. 3d, cryoEM density maps (in Ext. Data) should be referred to.

- In the figure text of Ext. Data Fig. 7a, it should be made clear that this concerns comparison of these structures to the Cas9 18-20MM kinked active Cas9 conformation.

**Author Rebuttals to First Revision:**

Referee #1:

The revised manuscript has been improved by the inclusion of new data--in particular, the cleavage result with the 7-aa mutant of the RuvC loop, which interacts with the mismatched nucleotides. The cleavage of 18-20 MM of DNA is greatly reduced, while there is no effect on the cleavage of the target DNA. This new data paves the way for an improved variant of Cas9 that is able to describe both targeted and non-targeted situations in the distal region of the PAM. I still have some minor concerns.

1. In the Cas9-12-14 MM DNA complex, Cas9 is similar in the linear and the kinked conformation, but the gRNA:TS duplex is different. The authors aligned these two structures in Fig. 2c, but this figure fails to clearly show the difference and similarity between the linear and the kinked structures. An additional Extended Data figure to superimpose the nucleic acids in these two complexes without protein may show the differences of the linear and kinked conformations.

To improve clarity of this figure we have adjusted Fig. 2c to depict the phosphate atoms of the nucleobase positions undergoing the conformational change in the PAM-distal duplex between the linear and kinked conformations. We believe that this aids clarity and conveys the conformational change appropriately.

2. In the Cas9-12-14 MM DNA complex, a linear and a kinked duplex conformation are resolved. However, the authors mention in line 79 that "Cas9 has the same conformation in the two kinked duplex structures (Figure 2)." What do these two kinked conformations refer to?

Thank you for noticing this mistake. This is a typo – Cas9 protein has the same conformation between the 12-14MM linear and 12-14MM kinked conformations (RMSD 1.9Å). This has now been fixed in the text.

3. The authors observed a product binding state for the RuvC domain, with nucleotides C18 and G19 positioned in its catalytic pocket. What about the other product containing nucleotides 1-17? The ssDNA substrate binding state with two Mg2+ of the RuvC domain of Cas12i2 were reported last year. Comparison of the substrate and product binding states of the RuvC domain may provide additional insight into the molecular mechanism of the RuvC domain.

Positions 1 – 8 of the other NTS product is still present in our map, as shown in extended data figure 4. These bases are largely held in place via a network of stacking and electrostatic interactions. Positions 9 – 17 are not visible in our map, most likely due to flexibility.

We thank the reviewer for this excellent point. Our catalytic mechanism is in overall agreement with that suggested for Cas12i2 and Cas12j (Pausch, 2021). These references have now been added to the text.

Referee #2:

The manuscript has been substantially improved, all concerns that I have raised were appropriately addressed - well done!!


Referee #3:

I would like to thank the authors for addressing my comments appropriately. 

I have some remaining minor remarks:

- The figure text of Fig. 1 panel says (b), but I think the text after it still belongs to panel (a).

Thank you for noticing this mistake. This typo has now been fixed.

- Panel labels in the (Ext. Data) figure text are sometimes capitalized, sometimes not.

- I appreciate that in Fig. 3d it is indicated that the 'map' is an isosurface presentation. However, for other figures also showing a 'map/density/surface' around the atoms, it should be stated in the figure text whether this is a cryoEM density map or an isosurface representation, and as for Fig. 3d, cryoEM density maps (in Ext. Data) should be referred to.

- In the figure text of Ext. Data Fig. 7a, it should be made clear that this concerns comparison of these structures to the Cas9 18-20MM kinked active Cas9 conformation.

These changes have been made.
